# Direct and Indirect Targeting of PP2A by Conserved Bacterial Type-III Effector Proteins

**DOI:** 10.1371/journal.ppat.1005609

**Published:** 2016-05-18

**Authors:** Lin Jin, Jong Hyun Ham, Rosemary Hage, Wanying Zhao, Jaricelis Soto-Hernández, Sang Yeol Lee, Seung-Mann Paek, Min Gab Kim, Charles Boone, David L. Coplin, David Mackey

**Affiliations:** 1 Department of Horticulture and Crop Science, The Ohio State University, Columbus, Ohio, United States of America; 2 Department of Plant Pathology, The Ohio State University, Columbus, Ohio, United States of America; 3 Department of Plant Pathology and Crop Physiology, Louisiana State University Agricultural Center, Baton Rouge, Louisiana, United States of America; 4 Division of Applied Life Science (BK21Plus), PMBBRC, Gyeongsang National University, Jinju daero, Jinju, Republic of Korea; 5 College of Pharmacy, Research Institute of Pharmaceutical Science, PMBBRC, Gyeongsang National University, Jinju daero, Jinju, Republic of Korea; 6 Department of Molecular Genetics, University of Toronto, Toronto, Ontario, Canada; 7 Department of Molecular Genetics, The Ohio State University, Columbus, Ohio, United States of America; Scottish Crop Research Institute, UNITED KINGDOM

## Abstract

Bacterial AvrE-family Type-III effector proteins (T3Es) contribute significantly to the virulence of plant-pathogenic species of *Pseudomonas*, *Pantoea*, *Ralstonia*, *Erwinia*, *Dickeya* and *Pectobacterium*, with hosts ranging from monocots to dicots. However, the mode of action of AvrE-family T3Es remains enigmatic, due in large part to their toxicity when expressed in plant or yeast cells. To search for targets of WtsE, an AvrE-family T3E from the maize pathogen *Pantoea stewartii* subsp. *stewartii*, we employed a yeast-two-hybrid screen with non-lethal fragments of WtsE and a synthetic genetic array with full-length WtsE. Together these screens indicate that WtsE targets maize protein phosphatase 2A (PP2A) heterotrimeric enzyme complexes via direct interaction with B’ regulatory subunits. AvrE1, another AvrE-family T3E from *Pseudomonas syringae* pv. tomato strain DC3000 (*Pto* DC3000), associates with specific PP2A B’ subunit proteins from its susceptible host Arabidopsis that are homologous to the maize B’ subunits shown to interact with WtsE. Additionally, AvrE1 was observed to associate with the WtsE-interacting maize proteins, indicating that PP2A B’ subunits are likely conserved targets of AvrE-family T3Es. Notably, the ability of AvrE1 to promote bacterial growth and/or suppress callose deposition was compromised in Arabidopsis plants with mutations of PP2A genes. Also, chemical inhibition of PP2A activity blocked the virulence activity of both WtsE and AvrE1 *in planta*. The function of HopM1, a *Pto* DC3000 T3E that is functionally redundant to AvrE1, was also impaired in specific PP2A mutant lines, although no direct interaction with B’ subunits was observed. These results indicate that sub-component specific PP2A complexes are targeted by bacterial T3Es, including direct targeting by members of the widely conserved AvrE-family.

## Author Summary

Gram-negative bacterial pathogens employ type-III effector (T3E) proteins to suppress host immunity and promote disease symptoms. AvrE-family T3Es, which are widely distributed among plant-pathogenic bacteria, suppress host defense responses and also contribute to water-soaking, which is perhaps the most common symptom of bacterial diseases and likely results in the release of nutrients from host cells to promote pathogen growth. Despite the central virulence functions of AvrE-family T3Es, their mode of action remains enigmatic largely due to their cell toxicity. We report here that two AvrE-family T3Es, WtsE from the maize pathogen *Pantoea stewartii* subsp. *stewartii* and AvrE1 from the tomato and Arabidopsis pathogen *Pseudomonas syringae* pv. tomato, each target protein phosphatase 2A (PP2A) complexes in susceptible hosts via direct interaction/association with specific B’ regulatory subunits. Chemical inhibitors were used to demonstrate that PP2A activity is required for the virulence functions of WtsE and AvrE1. PP2A isoform specificity was also tested using mutants of Arabidopsis. More generally, PP2A subunits regulate, both positively and negatively, rapid pattern-triggered immune responses in Arabidopsis. Thus, bacterial T3Es target sub-component specific PP2A complexes to manipulate host immunity and cause disease symptoms during infection.

## Introduction

Phytopathogenic bacteria encode effector molecules that remodel host cells in numerous ways [1–3]. One class of effectors is type-III effector proteins (T3Es) encoded by gram-negative bacteria and secreted into host cells through a hypodermic needle-like type-III secretion system (T3SS). In susceptible plants, T3Es target host proteins to induce disease symptoms, promote nutrient availability, and frequently suppress host immune responses through varied modes of action, including perturbation of host immune receptors, disruption of defense signal transduction, reprogramming of the host transcriptome, modulation of hormone signaling, perturbation of host metabolism, remodeling of cytoskeleton structure, and disruption of polarized secretion and cell wall reinforcement [4–8]. Collectively, the T3Es deployed by bacterial pathogens work in a ‘multifunctional, cooperative, and redundant’ manner to ensure successful perturbation of host defense networks [9]. However, functional redundancy between individual T3Es can explain why single gene knockouts of T3Es often have minimal effects on bacterial virulence.

The AvrE-family of T3Es are well conserved in a wide-spectrum of plant pathogenic bacteria, including the genera *Pseudomonas*, *Pantoea*, *Erwinia*, *Dickeya*, *Ralstonia* and *Pectobacterium*, with hosts ranging from monocots to dicots, including maize, Arabidopsis, tomato, apple and pear. AvrE-family T3Es make central, frequently essential, contributions to the virulence of these bacteria and are encoded by genes physically adjacent to the *hrp*/*hrc* gene cluster, which encodes the proteins that form the T3SS apparatus [1, 10–22]. DspA/E is an AvrE-family T3E from *Erwinia amylovora* (*Ea*), the fire blight pathogen of apple and pear trees. Unlike wild-type *E*. *amylovora*, a *dspA/E* mutant fails to cause necrosis and to suppress cell wall based defenses [13, 23]. Mutation of *PopS*, an ancient ortholog of AvrE-family T3Es, diminished the ability of *Ralstonia solanacearum* to cause bacterial wilt of tomato and potato [12]. DspE, the only known type-III effector encoded by the broad-host-range bacterial soft-rot pathogen *Pectobacterium carotovorum* subsp. *carotovorum* (*Pcc*), is required and sufficient for disease-associated cell death in host plants [18, 24]. Despite their importance for the virulence of bacteria infecting both model and crop plants, the mechanism by which AvrE-family T3Es perturb host cells is poorly understood.

*Pantoea stewartii* subsp. *stewartii* (*Pnss*) is a bacterial pathogen that causes Stewart’s wilt and leaf blight of maize by infecting the vasculature and leaves, respectively. Leaf blight is characterized by water-soaked (wts) lesions for which the AvrE-family T3E, WtsE, was named. Virulence of *Pnss* is vastly reduced by mutations of *wtsE* [20]. Furthermore, heterologous delivery of WtsE alone into leaf cells of host and non-host plants is sufficient to induce wts lesions. [10]. Additionally, WtsE suppressed plant defenses, including plant cell wall reinforcement, in Arabidopsis leaves [14]. The ability of WtsE to promote *Pnss* growth and disease symptoms in the leaves of maize seedlings relies on its ability to perturb the metabolism of aromatic compounds [8].

*Pseudomonas syringae* pv. tomato DC3000 (*Pto* DC3000) causes necrotic lesions on leaves of tomato and *Arabidopsis thaliana*. The *avrE1* gene, which encodes the AvrE-family T3E from *Pto* DC3000, is part of the conserved effector locus (CEL) adjacent to the *hrp/hrc* gene cluster. The *Pto* ΔCEL mutant is significantly diminished in its ability to multiply, cause disease symptoms, and suppress cell wall-based defense in leaves of both tomato and Arabidopsis [11, 13, 15]. In addition to AvrE1, the CEL locus encodes for another T3E, HopM1, which displays functional redundancy with AvrE1. *Pto* DC3000 *ΔavrE1* and *ΔhopM1* mutants induce fewer disease lesions on tomato leaves, but still grow as well as wild-type *Pto* DC3000 in tomato and in Arabidopsis [11]. Unlike these single mutants, an *ΔavrE1/ΔhopM1* double mutant is as severely impaired as *Pto* ΔCEL in its ability to induce disease symptoms and to grow in tomato and Arabidopsis leaves [11]. The virulence of the *Pto* ΔCEL mutant is restored by expression of either AvrE1 or HopM1 from a plasmid [13, 25]. Thus, AvrE1 and HopM1 make critical, functionally redundant contributions to the virulence of *Pto* DC3000 in Arabidopsis and tomato.

Identifying the targets of T3Es is a key step towards understanding their mode of action. HopM1 targets multiple Arabidopsis proteins for degradation through the plant proteasome [25]. Elimination of one of these targets, an ADP ribosylation factor (ARF)-guanine-nucleotide exchange factor (GEF) called AtMIN7, is postulated to suppress plant defenses by affecting the secretion of antimicrobial cargos [25]. The ability of HopM1 to eliminate AtMIN7 is blocked upon activation of resistance-proteins by other incompatible bacterial T3Es, indicating a mechanism by which effector-triggered immunity may reinforce basal plant defenses [26]. However, the exact consequence of HopM1 targeting AtMIN7 and other AtMINs remains unknown.

In contrast to HopM1, functionally validated targets of the AvrE-family T3Es remain unknown. Two lines of evidence support the hypothesis that AvrE-family T3Es will target related proteins in their various plant hosts. First, the ability of AvrE-family T3Es from one bacterial species to complement mutations of AvrE-family T3Es genes in other bacterial species indicates conserved functions between family members [10, 23]. Also consistent with a conserved mode of action of AvrE-family T3Es, mutation of conserved WxxxE motifs or of a putative C-terminal endoplasmic reticulum membrane retention/retrieval signal (ERMRS) disrupts the function of WtsE and AvrE1 in both host and non-host plants [21]. Based on the function of WxxxE motifs in other bacterial T3Es, AvrE-family effectors are hypothesized to mimic guanine nucleotide exchange factors (GEFs) [21, 24, 27–29].

Protein phosphatase 2A (PP2A), which is the major serine-threonine phosphatase in plants, is a heterotrimeric complex composed of an A subunit, a B subunit, and a catalytic C subunit [30]. The B subunits are a diverse group that play prominent roles in regulating the subcellular localization and substrate specificity of the holoenzyme. The Arabidopsis genome encodes 17 B subunits which, based on conserved domains/motifs in the encoded proteins, can be further divided into B (2 genes), B’ (9 genes), and B” (6 genes) subunits [31]. Expressed sequence tag analysis [32] and a protein database search (this study) indicate that the maize genome encodes at least 9 B’ subunit genes. Specific isoforms of PP2A, defined by their subunit composition, are required for vegetative and root growth, microtubule function, as well as various metabolic, stress, light, and hormone signaling pathways [33–36]. PP2A is also involved in plant defenses. Silencing PP2A C subunits in tobacco resulted in enhanced resistance against *P*. *syringae* [37]. PP2A dephosphorylates and thus destabilizes Arabidopsis type-I ACC synthase (ACS) enzymes [38]. As a result, constitutive and flg22-induced ethylene production are abnormal in an *rcn1* (*a1*) mutant, which disrupts the most abundant isoform of the A subunits and has lower overall PP2A levels. Mutation of the PP2A B’γ subunit resulted in constitutive activation of defense responses under low light [39]. PP2A isoforms containing A1, B’η/ζ, and C4 associates with and regulate the phosphorylation status of BRI1 associated kinase 1 (BAK1), which is a co-receptor for pathogen-associated molecular patterns (PAMPs), such as flagellin and EF-Tu, and for the hormone brassinosteroid. Thus, specific isoforms of PP2A attenuate early signaling in response to PAMPs [40].

Here we show that PP2A is a functionally significant molecular target of multiple AvrE-family T3Es. WtsE interacted with two maize PP2A B’ subunit proteins and arrested the growth of yeast cells dependent on PP2A. Similarly, AvrE1 associated with specific Arabidopsis PP2A B’ subunit proteins that are homologous to the maize WtsE-interacting proteins (WIPs). Moreover, AvrE1 also directly associated with the maize WIPs. Virulence activities of AvrE1 in *Nicotiana benthamiana* and WtsE in maize, including induction of disease symptoms and perturbation of metabolism, were attenuated by the PP2A-inhibitor cantharidin. Increased accumulation of AvrE1 in the presence of cantharidin confirmed the recently reported plasma membrane localization of the full-length effector protein. The ability of AvrE1 to promote *Pto* DC3000 growth and to suppress bacteria-induced cell wall reinforcement was dependent upon genes encoding specific Arabidopsis PP2A B’ subunits. The virulence function of HopM1 also depends on a specific subset of PP2A isoforms, presumably in an indirect fashion. More generally, PP2A A and B’ subunits appeared to make distinct contributions to early PAMP-signaling in Arabidopsis. These findings indicate that specific isoforms of PP2A are key regulators of plant defense that are targeted, directly or indirectly, by virulence-promoting T3Es.

## Results

### WtsE interacts with maize PP2A B’ subunit proteins in yeast-two-hybrid assays

**Fig 1:**
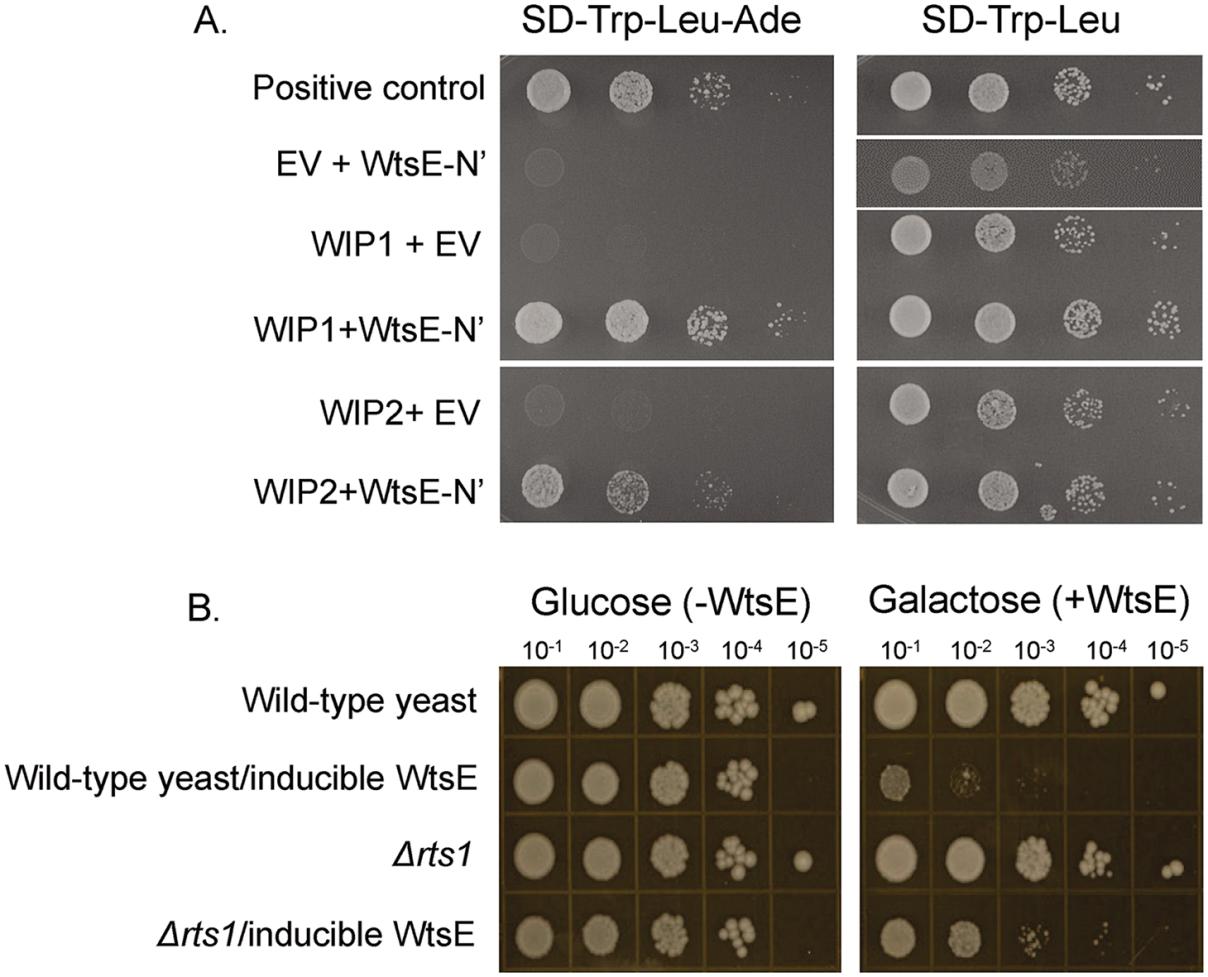
WtsE interacts with two maize PP2A B’ subunit proteins in yeast-two hybrid assays, and the deletion of the yeast PP2A B’ gene alleviates WtsE-induced cell death in yeast. (A) The N-terminal half of WtsE (WtsE-N’, aa 1–964) interacts with two maize PP2A B’ subunit proteins (WIP1 and WIP2). Interaction by yeast-two hybrid is selected on synthetic drop-out (SD) media lacking leucine, tryptophan, and adenine. EV: empty vector control. Pictures taken at 48 h are representative of three biological replicates. (B) Deletion of yeast *RTS1* (PP2A B’) allows better growth of yeast cells expressing full-length WtsE. WtsE expression is induced by galactose (2%), and is suppressed by glucose (2%). Pictures, taken at 96 hours after plating of a dilution series, show a representative of three biological replicates.

To identify targets of WtsE inside plant cells, we first screened for interacting maize proteins. We have previously shown that conditional expression of full-length WtsE, similar to AvrE1 and DspA/E, arrests the growth of *Saccharomyces cerevisiae* [14]. However, since yeast tolerates expression of the N-terminal (WtsE-N’, aa 1–964) or C-terminal (WtsE-C’, aa 964–1835) halves of WtsE, these fragments were used for yeast-two-hybrid (Y2H) screening against a cDNA library derived from young maize seedlings. Four major classes of WtsE-interacting maize proteins (WIPs) were identified. These included i) protein phosphatase 2A B’ regulatory subunits and ii) kinase domains of leucine-rich repeat receptor like kinases (LRR-RLKs) that interacted with the N-terminal half of WtsE and iii) ankyrin repeat domain proteins and iv) putative PRL1-interacting factor G proteins that interacted with the C-terminal half of WtsE (Table 1). Notably, DspA/E from *E*. *amylovora* has been shown to interact with the serine/threonine protein kinase domain of several apple LRR-RLKs designated as DIPMs (DspA/E-interacting protein of *Malus* ×*domestica*) [41]. WIP3, 4, and 5 each share protein sequence similarities with the four DIPMs ranging from 34% to 59% (Table 1). The role of PP2A and LRR-RLKs in plant immune functions, including the recognition of PAMPs and PAMP-induced defense signal transduction [40, 42], focused our attention on these two classes of WIPs. The interactions of WtsE-N’ with two maize PP2A B’ proteins (WIP1 and WIP2) and with three LRR-receptor kinases (WIP3, WIP4, and WIP5) have been confirmed by directed yeast-two hybrid assays (Fig 1A and S1A Fig).

**Table 1:** WtsE interacting proteins (WIPs) from yeast-two-hybrid screening.

Name	Y2H positive cDNA clones	Gene accession No.	Gene annotation	Closest homologs	Notes
WtsE-N’ interacting proteins					
WIP1	N1-1, N1-3	NM_001153392	Protein phosphatase 2A B’ regulatory subunit	AT3G09880: PP2A B’β (*At*); AAP68376.1 (*Os*)	nt 501–1059 of full-length (1569bp) cDNA
WIP2	N1-8, N1-9	NM_001155502	Protein phosphatase 2A B’ regulatory subunit	AT5G25510: PP2A B’ι (*At*); CAC85920: PP2A B’ι/κ (*Os*).	nt 90–909 of full-length (1103bp) cDNA
WIP3	N1-2, N1-4, N1-5, N1-6, N1-7	BT060755.1	Leucine-rich repeat receptor-like kinases	AT5G43020 (*At*); AT5G67200 (*At*); CAE04495 (*Os*).	cDNA clones contain the C’-terminal kinase domain.
					Protein sequence similarity with:
					DIPM1—50%
					DIPM2—38%
					DIPM3—49%
					DIPM4—34%
WIP4	N2-9	BT054783	Leucine-rich repeat receptor-like kinases	AT1G10850: LRR-RLK (*At*); NP_001048614 (*Os*).	Protein sequence similarity with:
					DIPM1—41%
					DIPM2—36%
					DIPM3—40%
					DIPM4—36%
WIP5	N2-18	BT088209	Leucine-rich repeat receptor- like kinases	AT1G48480: Receptor-like kinase 1 (*At*); AAN05336 (*Os*).	Protein sequence similarity with:
					DIPM1—40%
					DIPM2—59%
					DIPM3—36%
					DIPM4—57%
WtsE-C’ interacting proteins					
WIP6	C1-1, C1-4, C1-5, C1-6, C1-7	NM_001175852	Ankyrin repeat family protein	AT2G44090 (*At*); AT3G59910 (*At*).	Weak homology to AAD41595: *Pristionchus pacificus* CED-3 protein
WIP7	C1-3, C1-9, C1-10	NM_001154512	Ankyrin domain-containing proteins	AT5G07270: E3 ubiquitin-protein ligase XBAT33 (*At*); BAC98626: Xa21-binding protein 3-like (*Os*).	
WIP8	C1-8	NM_001152611	Hypothetical protein	AT2G13690: putative PRLI-interacting factor (*At*); NP_001052356: Similar to PRLI-interacting factor G (*Os*).	

DIPM: DspA/E-interacting protein of *Malus* ×*domestica*. DIPM sequences were retrieved from Meng et al., 2006 [41]. WIP3, 4, and 5 protein sequences were aligned with the four DIPMs using BlastP algorithm on NCBI (http://blast.ncbi.nlm.nih.gov/).

### PP2A contributes to WtsE-mediated growth arrest in yeast

In addition to the Y2H screening with each half of WtsE, we also performed a synthetic genetic array (SGA) screen in *S*. *cerevisiae* [43]. Full-length WtsE was conditionally expressed in an ordered array of ~5000 viable yeast gene deletion mutants (collectively accounting for ~80% of all yeast genes). In wild-type yeast, expression of full-length WtsE arrested growth. The screen identified mutations that permitted yeast to better tolerate the expression of WtsE (potential targets of WtsE or transducers of its virulence function) and mutations that exacerbated the detrimental phenotype of WtsE expression (potential suppressors of WtsE function). Among those that allowed better growth of the WtsE-expressing yeast strains were mutations of four genes related to PP2A (*ppm1*, *rts1*, *rrd2* and *pph21*, Table 2). *PPH21* encodes one of three yeast PP2A catalytic subunits. *RTS1* encodes for a PP2A B’ subunit, which is one of the two yeast B-type subunits. *PPM1* and *RRD2* encode proteins essential for the PP2A complex assembly and activation to phospho-serine/threonine specificity, respectively [44, 45]. The role of RTS1 in mediating WtsE-induced growth arrest was confirmed by targeted deletion of *RTS1* in wild-type and WtsE-expressing yeast strains (Fig 1B). The results of the Y2H and SGA screens led us to hypothesize 1) that WtsE disrupts yeast growth by targeting PP2A via direct interaction with the B’ regulatory subunit and 2) that WtsE promotes *Pnss* virulence by similarly targeting PP2A via B’ subunits *in planta*.

**Table 2:** Partial list of mutations that permit yeast to better tolerate WtsE expression.

Orf Name	Gene	Gene Function	Size Difference (Ctrl mean-Expt mean)
PP2A-related genes			
YDR435C	*PPM1* *	C-terminal protein carboxyl methyltransferase activity	-1.283027
YOR014W	*RTS1*	Protein phosphatase type 2A activity	-0.648948
YPL152W	*RRD2*	Peptidyl-prolyl cis-trans isomerase activity	-0.555954
YDL134C	*PPH21*	Protein phosphatase type 2A activity	-0.553447
Sphingolipid biosynthesis-related genes			
YCR034W	*FEN1* * ^+^	Fatty acid elongase activity	-1.228157
YDR497C	*ITR1* ^+^	Myo-inositol transmembrane transporter activity	-1.200737
YGR143W	*SKN1* ^+^	Glucosidase activity	-0.965214
YDR072C	*IPT1* ^+^	Transferase activity, transferring phosphorus-containing groups	-0.763681
YEL042W	*GDA1* ^+^	Guanosine-diphosphatase activity	-0.541537
YPL057C	*SUR1*	Mannosyltransferase activity	-1.261966
YDR297W	*SUR2*	Sphingosine hydroxylase activity	-0.784637
YHL003C	*LAG1*	Sphingosine N-acyltransferase activity	-0.636515

Ctrl mean: colony size (pixel area) of Gus1-expressing yeast double mutant strain. Expt mean: colony size (pixel area) of WtsE-expressing yeast double mutant strain. Colony size is normalized by dividing each colony size value by the mean colony size across a particular plate from which the colony was derived.

* indicates hits called by both automated and manual scoring. All other hits are from automated scoring only.

^+^ indicates genes whose products contribute to DspA/E-induced cell death in yeast [46]. For a full list of SGA results, refer to S1 Table.

DspA/E was recently reported to arrest yeast cell growth by depletion of long chain bases (LCBs) via disruption of sphingolipid biosynthesis [46]. Growth of yeast expressing DspA/E was restored by mutation of genes encoding enzymes that convert the LCB-precursors phytosphingosine (PHS) and dihydrosphingosine (DHS) into various ceramides, apparently at the expense of LCB production. In our SGA screening, eight of the mutants that better tolerated WtsE expression had mutations in the sphingolipid biosynthetic pathway, including *fen1*, *itr1*, *skn1*, *ipt1*, *gda1*, *sur1*, *sur2*, and *lag1* (Table 2). As reported for the DspA/E-expressing yeast cells, we also found that the growth of WtsE-expressing yeast cells was partially restored by supplementation with 15 μM PHS or DHS (S1B Fig).

### Cantharidin inhibits the virulence function of WtsE in maize seedlings

**Fig 2:**
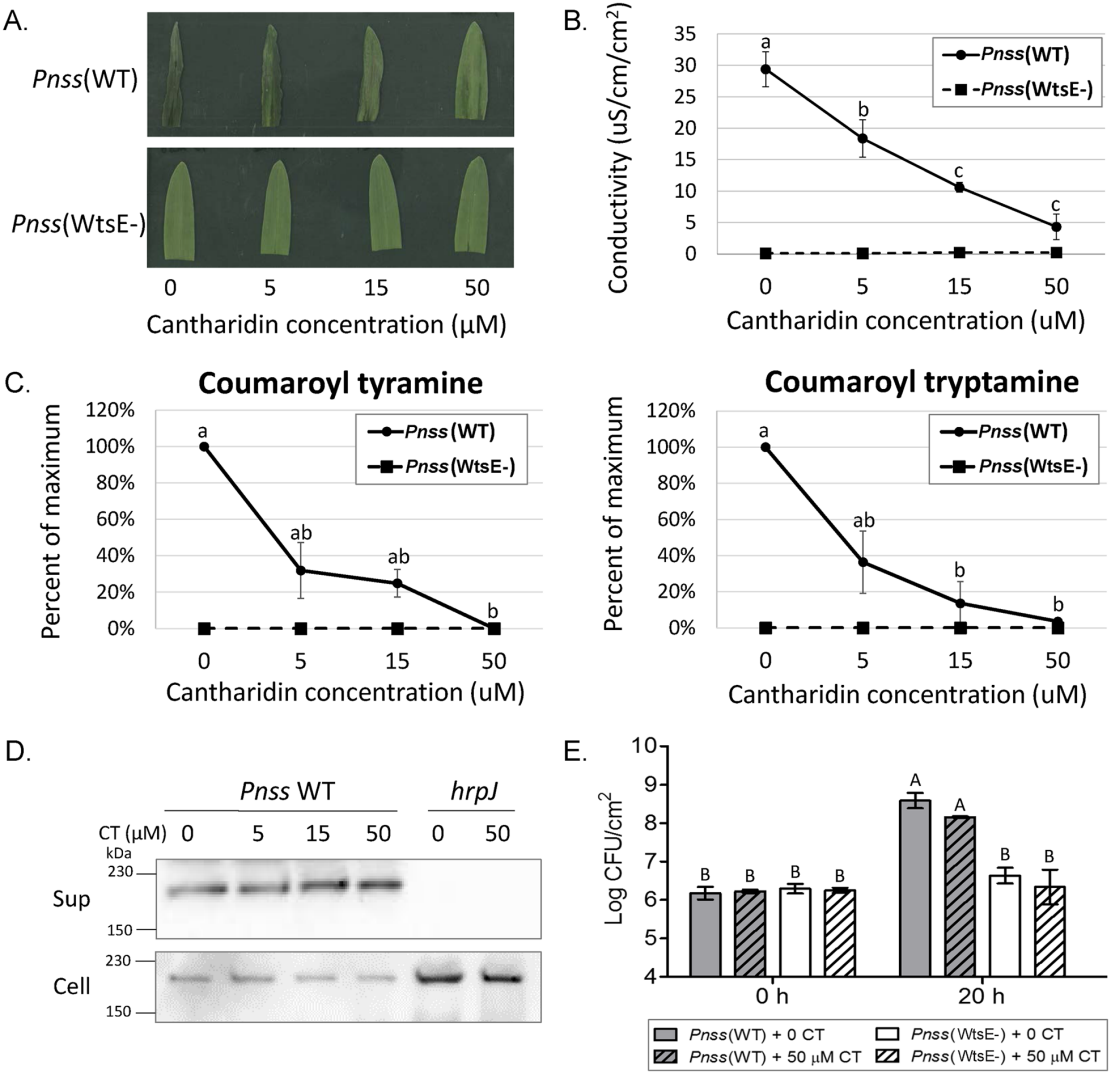
Virulence activities of WtsE in maize seedling leaves are suppressed in a dose dependent manner by cantharidin independent of an effect on WtsE secretion or short-term *Pnss* growth. Visual cell death symptoms (A) and electrolyte leakage (B) induced by wild-type, but not the *wtsE* mutant, *Pnss* are suppressed by cantharidin. Six-day-old maize seedlings were vacuum infiltrated with 10^9^ CFU/mL of wild-type *Pantoea stewartii* subsp. *stewartii* (*Pnss* WT) or a *wtsE* mutant strain (*Pnss* WtsE-) supplemented with 0, 5, 15, or 50 μM cantharidin. Similarly treated samples were subjected to electrolyte leakage analysis (B) or metabolite measurements (C). Values shown in (B) are mean ± SD from three biological replicates and were analyzed by one-way ANOVA followed by Tukey test. Different letters indicate significant differences at P<0.05. (C) The ability of wild-type, but not *wtsE* mutant, *Pnss* to induce accumulation of conjugated derivatives of phenolic amino acids is suppressed by cantharidin. Quantities of coumaroyl-tyramine and coumaroyl-tryptamine were measured by LC-MS/MS, normalized to the internal control formononetin and quantified relative to standard curve generated from pure coumaroyl-tyramine or coumaroyl-tryptamine. Values shown are mean ± SD of relative quantities of each compound with 100% representing the amount induced by *Pnss* WT in the absence of cantharidin. The data is compiled from three biological replicates and was analyzed by one-way ANOVA followed by Tukey test. Different letters indicate significant differences at P<0.05. (D) Cantharidin does not affect production or secretion of WtsE from *Pnss*. Wild-type *Pnss* and the type-III secretion system deficient mutant (*hrpJ*) bacteria were grown in *hrp*-inducing liquid medium supplemented with 0, 5, 15, or 50 μM cantharidin (CT). Cultures were separated into supernatant (Sup) and cell (Cell) fractions by centrifugation. The WtsE protein (~204 kDa) in each fraction was detected by immunoblotting using an anti-DspA/E antibody. Shown is a representative blot from three biological replicates. (E) Cantharidin treatment does not affect short-term *Pnss* growth in maize seedlings following high titer infiltration. Six-day-old maize seedlings were vacuum infiltrated with 10^9^ CFU/mL of wild-type *Pantoea stewartii* subsp. *stewartii* (*Pnss* WT) or a *wtsE* mutant strain (*Pnss* WtsE-) supplemented with 0 or 50 μM cantharidin (CT). Bacterial growth was assessed immediately following infiltration (0 h), or at 20 hai. Shown are mean ± SD from 3 biological replicates and data were analyzed by one-way ANOVA followed by Tukey test. Different letters indicate significant difference at P<0.01.

To test the hypothesis that PP2A activity is required for the virulence activity of WtsE *in planta*, we used cantharidin, which is a toxin known to inhibit PP2A and, with lower potency, PP1. Suspensions of wild-type and *wtsE* mutant strains of *Pnss* were supplemented with different concentrations of cantharidin (0, 5, 15, or 50 μM) and vacuum-infiltrated into 6-day old maize seedlings. While cantharidin had no visible effect on the seedlings infiltrated with the *wtsE* mutant, it inhibited, in a dose-dependent manner, the WtsE-dependent necrotic symptoms induced by wild-type *Pnss* (Fig 2A). In a quantitative assay, measurements of electrolyte leakage from the infiltrated leaf area confirmed that cantharidin inhibits disease-associated cell death induced by WtsE (Fig 2B). The virulence activity of WtsE in maize seedling leaves has been linked to alterations of phenylpropanoid metabolism marked by accumulation of coumaroyl-amino acid conjugates: coumaroyl tyramine (CouTyr [8]) and coumaroyl tryptamine (CouTrp) (Fig 2C). We tested by liquid chromatography-tandem mass spectrometry (LC-MS/MS) the effect of cantharidin on WtsE-induced accumulation of CouTyr and CouTrp. In accordance with the symptom development, the accumulation of both compounds was inhibited by cantharidin in a dose-dependent manner (Fig 2C).

Two experiments were done to control for the effects of cantharidin. First, an *in vitro* secretion assay was performed to determine if cantharidin inhibited WtsE production and/or secretion from *Pnss*. When *Pnss* was cultured in a *hrp*/*hrc*-inducing medium, supplementation of the medium with various concentrations of cantharidin (0, 5, 15, or 50 μM) affected neither the production of the WtsE protein nor its secretion into the culture medium (Fig 2D). Second, the *in planta* population of *Pnss* was measured to determine if cantharidin affects the viability or growth of the bacteria following the high titer infiltrations used in Fig 2. Cantharidin induces defense responses in a number of plant species [34], though it is not known if this occurs in maize seedlings. The presence of 50 μM cantharidin did not significantly affect the number of viable *Pnss* cells 20 hai into maize seedlings (Fig 2E). Thus, although cantharidin inhibits the ability of WtsE to induce metabolic perturbations, cell death, and disease symptoms (Fig 2A–2C), it does not inhibit the ability of WtsE to promote *Pnss* growth and/or maize defense responses in a way that alters *Pnss* growth during the first 20 hours of infection under these experimental conditions. Though we cannot rule out effects of cantharidin on PP1, the results of Fig 2 support our hypothesis that PP2A is a virulence target of WtsE.

### AvrE1 associates with PP2A B’ subunit proteins including Arabidopsis homologues of the maize WIPs and the maize WIPs

**Fig 3:**
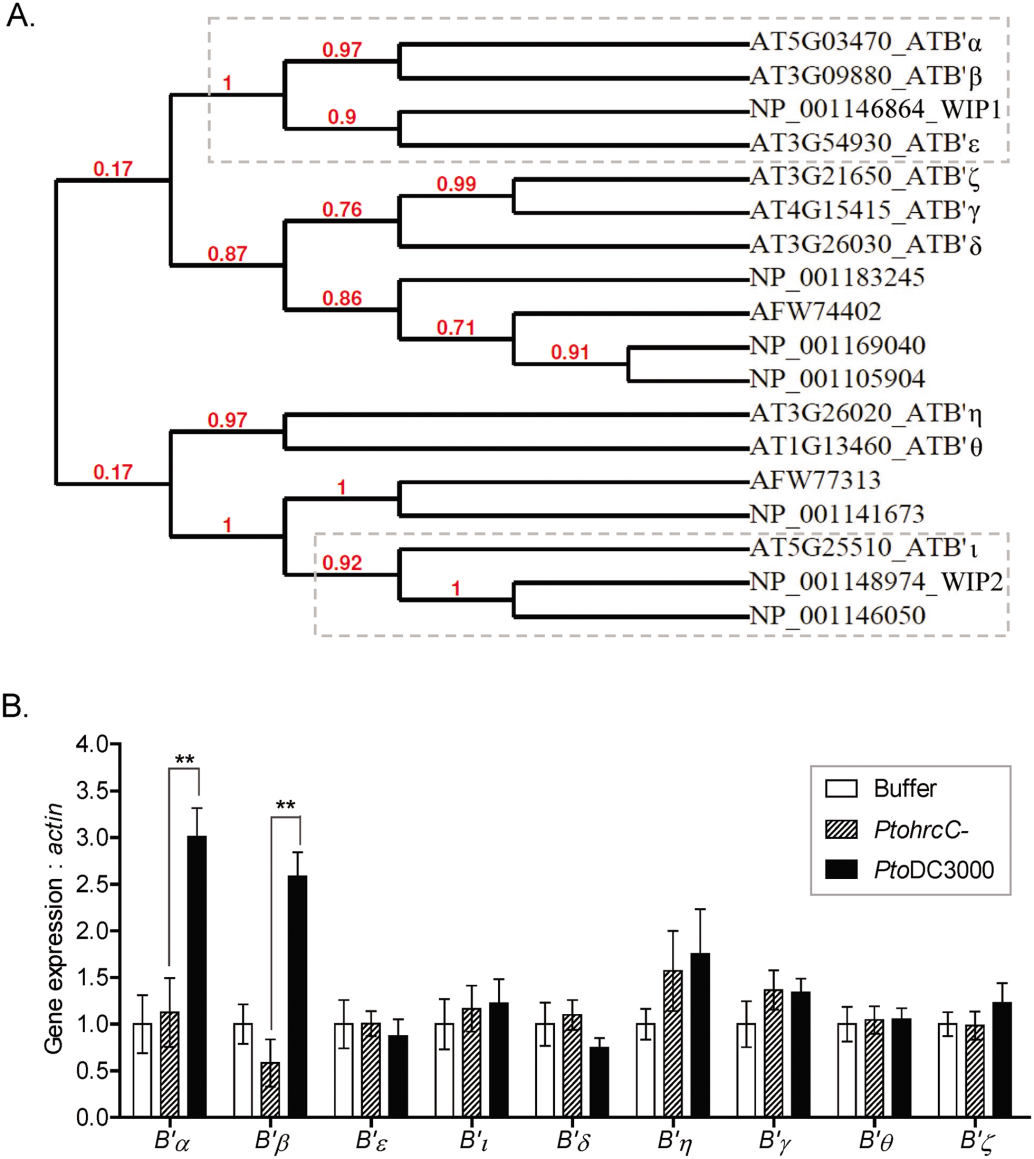
Transcripts of specific Arabidopsis PP2A B’ regulatory subunit are induced upon *Pto* DC3000 infection dependent on T3SS. (A) Protein phylogeny of Arabidopsis and maize PP2A B’ subunit proteins, including WIP1 and WIP2. Accession numbers starting with “AT” are Arabidopsis proteins; accession numbers starting with “NP” or “AFW” are maize proteins. (B) Expression of a subset of Arabidopsis B’ regulatory subunits is induced by *Pto* DC3000, but not by the type III secretion-deficient mutant *Pto hrcC-*. Five-week-old Col-0 Arabidopsis leaves were infiltrated with the indicated bacterial strains (10^8^ CFU/ml) or buffer (10 mM MgCl2). Samples were collected at 9 hai and subjected to qRT-PCR. Values shown are mean ± SD of normalized data from three biological replicates with transcript level in buffer treated samples set to 1. ** indicates a significant difference by Student’s t-test at P<0.01.

Since AvrE1 and WtsE are homologous effector proteins that share similar virulence functions in their respective host plants, we hypothesize that they may target related host proteins. This is supported by our observation that expression of WtsE in the *Pto* ΔCEL mutant restored its virulence in Arabidopsis (*i*.*e*., inducing cell death, promoting bacterial growth, and suppressing callose deposition) to the same degree as does AvrE1 (S2 Fig). Therefore, based on our findings with WtsE, we hypothesized that AvrE1 also targets PP2A B’ subunits of *Arabidopsis thaliana* that participate in plant defense responses and/or bacterial pathogenesis. To begin exploring this hypothesis, we first generated a protein-sequence based phylogeny of the nine known Arabidopsis PP2A B’ subunits and nine predicted maize B’ subunits (Fig 3A, for maize B’ subunit prediction, refer to S1 Text Supplemental Material and Methods). Arabidopsis B’α, β, and ξ are the closest homologs of WIP1, and B’ι, is the closest homolog of WIP2. For further study in interaction assays with AvrE1, we selected these four Arabidopsis homologs of WIP1 and WIP2. We also tested the transcript levels of all nine Arabidopsis PP2A B’ subunit genes in response to *Pto* DC3000 infection. Compared to buffer or a *Pto hrcC-* mutant strain (a T3SS deficient mutant), *Pto* DC3000 infection induces expression of B’*α* and *β* but not any of the other B’ subunits (Fig 3B), indicating a potential role of these subunits in plant defense and/or bacterial pathogenesis.

**Fig 4:**
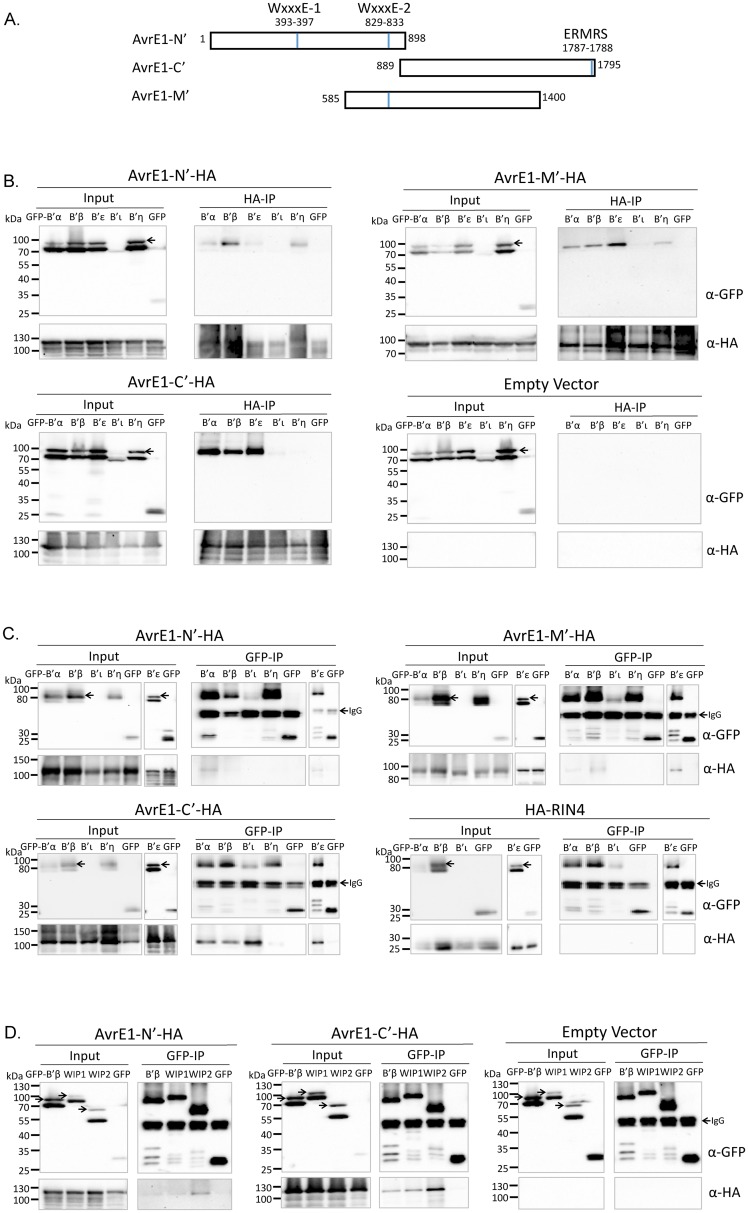
Non-lethal fragments of AvrE1 associate with Arabidopsis PP2A B’ regulatory subunits homologous to the WtsE-interacting proteins, as well as maize WIPs. (A) Diagram of non-lethal fragments of AvrE1 effector protein. WxxxE motifs and the putative ERMRS motif are indicated by blue lines. (B-C) AvrE1 fragments associate with specific Arabidopsis PP2A B’ subunits following *Agro*-transient expression in *N*. *benthamiana*. GFP-B’α, β, ξ, ι, η, or free GFP were co-expressed with AvrE1-HA fragments in *N*. *benthamiana*. Co-immunoprecipitation of GFP-B’α, β, ξ, ι, η, or free GFP was detected using anti-GFP antibody following HA-pull down of AvrE1 fragments (B). Reciprocally, PP2A B’ subunits were pulled-down using anti-GFP antibody and co-immunoprecipitation of AvrE1-HA fragments or HA-RIN4 was detected by immunoblotting with anti-HA antibody (C). Shown are representative blots from three biological replicates. GFP-B’ ξ in (C) was tested separately from the rest of the panel. (D) AvrE1 fragments associate with maize WIPs. Arabidopsis GFP-B’β, maize GFP-WIP1, GFP-WIP2, or free GFP were co-expressed with AvrE1-HA fragments in *N*. *benthamiana*. PP2A B’ subunits from Arabidopsis and maize were pulled down using anti-GFP antibody and co-immunoprecipitation of AvrE1-HA fragments was detected by immunoblotting with anti-HA antibody. Shown is a blot representative of three biological replicates, Arabidopsis GFP-B’β was included in two replicates. Arrows indicate predicted sizes of the GFP-PP2A B’ full-length constructs.

Using full-length AvrE1 to study protein-protein interactions in yeast and *in planta* is technically challenging because, similar to WtsE and DspA/E, AvrE1 induces yeast growth arrest and plant cell death [11, 47]. To circumvent this obstacle, we generated non-lethal fragments of AvrE1; an N-terminal half (AvrE1-N’, aa 1–898), a C-terminal half (AvrE1-C’, aa 889–1795), and a middle fragment that overlaps the split site between the N- and C-terminal halves (AvrE1-M’ aa 585–1400, Fig 4A). The middle fragment was chosen based on domain prediction of AvrE1 [GlobPlot, [48]] with the breakpoints chosen at regions with low complexity. Y2H experiments failed to reveal interactions between these AvrE1 fragments and Arabidopsis PP2A B’ subunits. As an alternative, we turned to *in planta* interaction assays.

In order to select appropriate control proteins, we first sought to determine the localization of AvrE-fragments and B’ subunits expressed in *N*. *benthamiana*. Confocal microscopy and membrane fractionation indicated that GFP-AvrE1-N’ (and AvrE1-N’-HA) and GFP-AvrE1-M’ (and AvrE1-M’-HA) were primarily plasma membrane localized and GFP-AvrE1-C’ (and AvrE1-C’-HA) were nuclear and plasma membrane localized (S3 Fig). Given their predominant plasma membrane localization, Arabidopsis RIN4 was selected as a control protein for the AvrE1-fragments in a subset of the experiments [49, 50]. We similarly tested the sub-cellular localization of GFP-tagged PP2A B’α, β, ξ, ι, and η subunits expressed in *N*. *benthamiana* (S3 Fig). B’α, β, ι, and η were nuclear and plasma membrane localized. B’ξ is associated with the membrane fraction, and shows intra-cellular speckles. Given its similar localization, B’η was selected as a properly localized control protein for analysis of B’α, β, ξ, and ι.

Association between Arabidopsis B’ subunits and fragments of AvrE1 was tested by reciprocal co-immunoprecipitation (Co-IP) following *Agrobacterium*-mediated transient expression in *N*. *benthamiana*. Pull-down of AvrE1-HA fragments followed by detection of GFP-B’ subunit fusions, or vice versa, revealed specific associations (Fig 4). AvrE1-C’ fragment strongly and reproducibly associated with Arabidopsis PP2A B’α, β, and ξ, but not with B’η or free GFP. Weak association with B’ ι was observed in two out of three replicates when AvrE1-C’-HA was being pulled down, presumably due to low expression of the GFP-B’ι fusion construct (Fig 4B). Reciprocal co-immunoprecipitation revealed apparent association with GFP-B’ι when the fusion protein was enriched in the GFP-IP fraction (Fig 4C). The AvrE1-N’ and M’ fragments showed weaker and more variable associations (Fig 4B and 4C). AvrE1-N’-HA and AvrE1-M’-HA associated with B’α, β and ξ in both co-immunoprecipitation directions. And when AvrE1 fragments were being pulled down, GFP-B’η was also detected to associate with AvrE1-N’ and -M’ fragments in two out of three experiments. The associations in HA-IP between GFP-B’ξ and AvrE1-N’-HA was also observed in two out of three replicates. HA-RIN4 did not interact detectably with free GFP or with any of the GFP-fused PP2A B’ proteins tested in GFP-pull down, indicating that non-specific pull down of plasma membrane proteins does not account for the associations observed with this assay (Fig 4C). We also tested whether mutations in the ERMRS motif at the C-terminus of AvrE1 [21] affects its association with the PP2A B’ proteins. The k1k2 (KK1787-88AA in full length AvrE1,) mutation altered neither the subcellular distribution of the AvrE1-C’ fragment nor its association with PP2A B’ α, β or ι (S4B Fig). The k1k2 mutant of AvrE1-C’ did associate weakly with the PP2A B’η subunit, indicating that the ERMRS motif may play a role in determining the specificity of PP2A subunit associations. Finally, to test the hypothesis that AvrE1 and WtsE have conserved virulence targets across plant species, we tested whether AvrE1 fragments and maize WIPs co-immunoprecipitate when transiently expressed in *N*. *benthamiana*. Pull down of GFP-WIPs constructs revealed similar association patterns with AvrE1 as their Arabidopsis PP2A B’ homologs. Specifically, AvrE1-C’-HA strongly, and AvrE1-N’-HA more weakly, associated with both WIP1 and WIP2 (Fig 4D).

As a second approach to test for associations between B’ subunits and fragments of AvrE1, we used bi-molecular fluorescence complementation (BiFC, S4A Fig). BiFC constructs fusing the N-terminal and C-terminal fragments of GFP (GFP^n^ and GFP^c^, respectively) to each fragment of AvrE1 and to PP2A B’α, β, ι, and η were constructed and tested for auto-active GFP reconstitution following co-expression in *N*. *benthamiana* with the corresponding free GFP^n^ or GFP^c^ construct. The AvrE1-M’ fragment displayed auto-activity when fused to either half of GFP and was therefore eliminated from these analyses. Subsequent tests with constructs lacking auto-activity confirmed the co-IP result that the AvrE1-C’ fragment associated specifically with PP2A B’α, β, and ι, but not with B’η. BiFC also indicated association of AvrE1-N’ with PP2A B’α, β, and ι, confirming the co-IP results for B’α and β and also indicating that AvrE1-N’ additionally associates with B’ι. Consistent with the localization patterns of the AvrE1 fragments and the PP2A B’ subunits, each of the BiFC associations appears to occur at the cell periphery. Based on the results of our various association assays, we posit that AvrE1 contains multiple domains that associate with plant PP2A B’ subunit proteins and that the C-terminal half of AvrE1 is responsible for maintaining strong and specific associations.

### Cantharidin inhibits AvrE1-induced cell death in *Nicotiana benthamiana*

We hypothesized that AvrE1, similar to WtsE, may require PP2A activity for its virulence function. Full-length AvrE1 induces potent cell death following its *Agro*-transient expression in *N*. *benthamiana* [11]. We tested whether co-infiltration of cantharidin with *Agrobacterium* delivering full-length AvrE1 could inhibit the cell death induced by AvrE1 in *N*. *benthamiana*. *Agrobacterium* strains that deliver *35S*::*GFP-AvrE1* (or an empty vector *35S*::*GFP*) were co-infiltrated with 0, 0.3, 1, 3, or 5 μM cantharidin and leaf samples were tested for cell death and protein expression. Unlike maize seedlings that have a higher tolerance to cantharidin, infiltration of 3 and 5 μM cantharidin induced macroscopic cell death in *N*. *benthamiana* in two and four out of four experiments, respectively. The intermediate and high levels of cell death caused by 3 and 5 μM cantharidin were confirmed by electrolyte leakage measurements in leaves expressing free GFP (S5C Fig). The protein levels of free GFP indicated that the higher concentrations of cantharidin also inhibit the *Agro*-mediated transient expression (S5A Fig). Despite the ability of cantharidin to induce cell death and inhibit *Agro*-mediated transient expression of GFP in leaves receiving *35S*::*GFP*, it reduced cell death and allowed higher GFP-AvrE1 expression in leaves receiving *35S*::*GFP-AvrE1* (S5A Fig). At 24 hai with concentrations of 0.3 and 1 μM cantharidin, expression of GFP-AvrE1 was apparent by western blotting. The phenotypic expression of GFP-AvrE1 in the absence of cantharidin is apparent by the cell death symptoms. However, despite 1) higher expression of AvrE1 with 0.3 and 1 μM cantharidin and 2) the tendency of cantharidin to cause cell death in *N*. *benthamiana* leaves, cell death in these samples was delayed from 24 to 32 hai (S5 Fig).

We also tested HopQ1, a T3E from *Pto* DC3000 that causes a hypersensitive response (HR, plant-defense-associated cell death) in *N*. *benthamiana*. Application of 1 μM and 3 μM of cantharidin also inhibits the *Agro*-transient expression of the *35S*::*GFP-HopQ1-1* fusion protein. However, cantharidin treatment accelerated HopQ1-1-induced cell death despite the low protein accumulation (S6 Fig). This observation is consistent with a previous report that silencing of PP2A C subunits in *N*. *benthamiana* exacerbates HR-type cell death [37]. The contrasting phenotype between AvrE1 and HopQ1 indicates that AvrE1-induced cell death in *N*. *benthamiana* is not likely an HR-type cell death. Rather, we hypothesize that the ability of cantharidin to suppress the cell death inducing activity of AvrE1 results from its inhibition of the AvrE1 virulence target, PP2A.

The use of cantharidin allowed *Agro*-transient expression of GFP-fused full-length AvrE1 in *N*. *benthamiana*. Attempts to detect association of PP2A B’ subunits with full-length GFP-AvrE1 failed (S7 Fig), possibly due to the low level accumulation of GFP-AvrE1 in the 24 hours prior to tissue collapse. However, GFP-AvrE1 did accumulate to levels sufficient for visualization under the confocal microscope. Consistent with the localization patterns of the AvrE1-N’, -M’, and -C’ fragments, GFP-AvrE1 appeared to be localized to the cell periphery. Biochemical fractionation of leaves in which *Agrobacterium* was co-infiltrated with 0.3 μM cantharidin, followed by immuno-precipitation and immunoblotting, indicated that GFP-AvrE1 is associated with microsomal membranes (S5A and S5B Fig). Consistent with another recent report [51], these data indicate that GFP-AvrE1 localizes to the plasma membrane of *N*. *benthamiana* cells.

### Specific PP2A isoforms are required for AvrE1 and HopM1 function in Arabidopsis

To study the role of specific PP2A B’ proteins, we obtained Arabidopsis T-DNA insertion lines for six of the nine PP2A B’ subunits: *b’α*, *β*, *η*, *ϒ*, *ϑ*, and *ζ*. In addition, two PP2A A subunit mutants (*rcn1-6*, PP2A *a1*; and *a2a3*, PP2A *a2* and *a3* double mutant) were also obtained to study the role of the holoenzyme (S8 Fig). Notably, we were unable to identify a knock-out or knock-down line for the AvrE1-associating B’ι or B’ξ subunits of PP2A. Using this collection of Arabidopsis lines, we sought to determine how the lack of specific A or B’ subunits affects the function of AvrE1 and HopM1.

**Fig 5:**
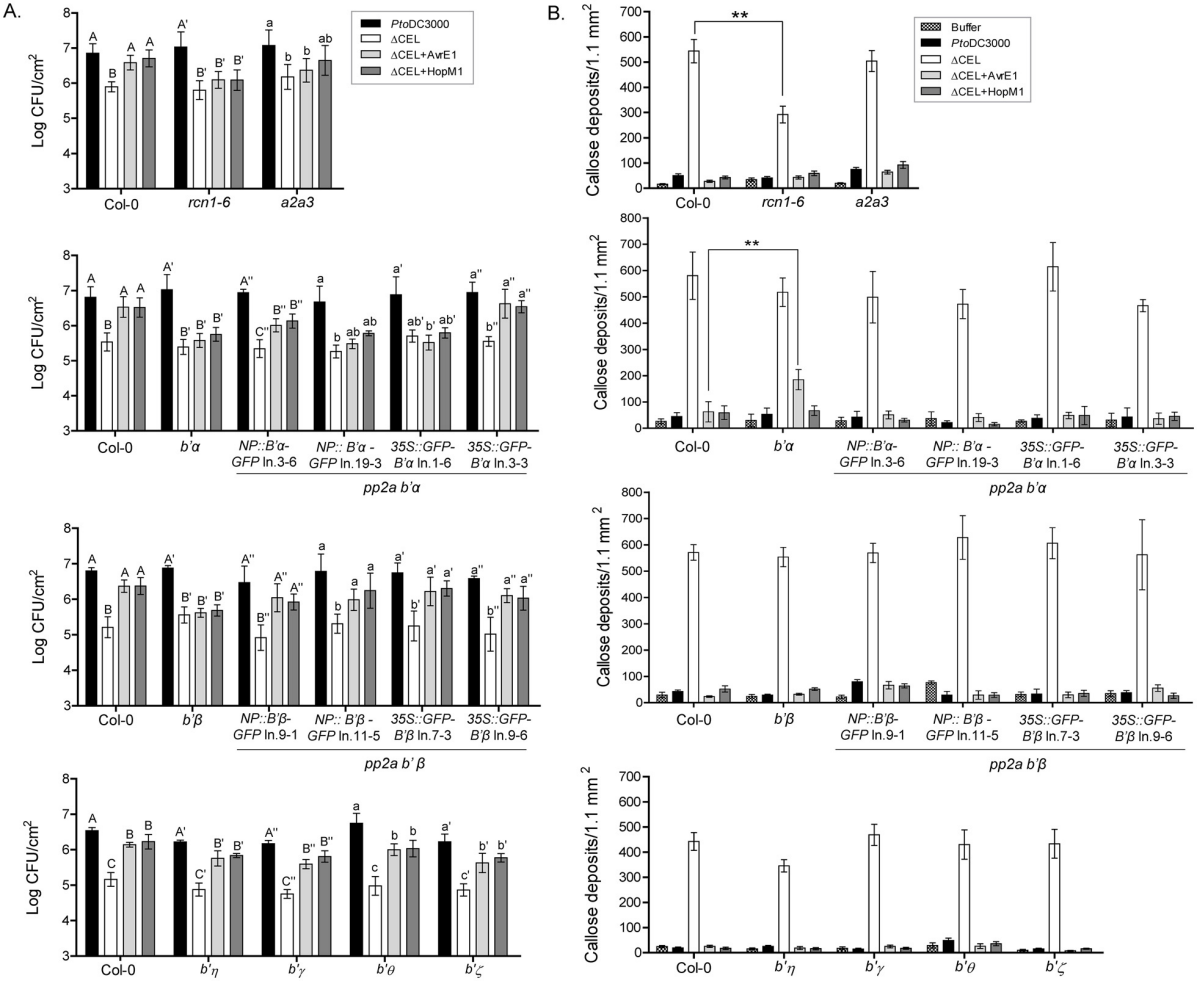
Specific PP2A B’ subunits are required for virulence activities of AvrE1 and HopM1 in Arabidopsis leaves. (A) Growth analysis in 5-week-old Arabidopsis plants, including PP2A mutants and complementation lines, at 4 days after infiltration of 10^5^ CFU/ml of *Pto* DC3000, ΔCEL, ΔCEL+AvrE1, and ΔCEL+HopM1. Values shown are mean ± SEM from four biological replicates for A subunit and *b’η*, *γ*, *θ*, and *ζ* mutants, from three biological replicates for complementation lines, and from at least six biological replicates for *α* and *β* mutants. The inability of *NP*::*α-GFP* ln.19-3 and *35S*::*GFP-α* ln. 1–6 to complement T3E-promoted bacterial growth may result from an adverse effect of too high and too low *α* transcript level, respectively (S8 Fig). Data was analyzed by one-way ANOVA followed by the Tukey test comparing different bacterial strains on individual plant genotypes. Different letters of the same style (eg. A vs B, or A’ vs B’) indicate significant differences at P<0.05 within the same plant genotype. (B) Suppression of *Pto* ΔCEL-induced callose deposition by AvrE1 depends on the PP2A B’*α* subunit. *rcn1-6* showed reduced callose deposition in response to ΔCEL infection. Leaves of five-week-old Arabidopsis plants were infiltrated with the indicated bacterial strains (10^8^ CFU/ml) or buffer (10 mM MgCl2), and collected at 16 hai for callose staining. Values shown are mean ± SEM from 3–5 biological replicates with Col-0 included in each individual experiment. ** indicates a significant difference by Student’s t-test at P<0.01.

First we measured the growth of *Pto* DC3000 and *Pto* ΔCEL, which lacks the genes encoding both AvrE1 and HopM1, as well as the plasmid complemented strains, termed ΔCEL+AvrE1 and ΔCEL+HopM1 (Fig 5A). AvrE1, expressed by the ΔCEL+AvrE1 strain, failed to restore the growth of *Pto* ΔCEL in *rcn1-6*, *a2a3*, and B’ *α* and *β* mutant plants. HopM1, expressed by the ΔCEL+HopM1 strain, failed to restore the growth of *Pto* ΔCEL in *rcn1-6* and B’ *α* and *β* mutant plants. AvrE1 and HopM1 were each able to complement the growth of *Pto* ΔCEL in wild-type plants and the other tested PP2A B’ subunit mutants (*b’η*, *ϒ*, *ϑ*, or *ζ*). Thus, AvrE1 and HopM1 require high level expression of PP2A A subunits and specifically B’ α and β containing isoforms of PP2A to promote the growth of *Pto* ΔCEL. We considered that low expression of AvrE1 and HopM1 from the plasmids carried by *Pto* ΔCEL might contribute to their requirement for specific PP2A B’ subunits. This appears not to be the case since the growth of strains with genomic deletions of *avrE1* (Δ*avrE1*), *hopM1* (Δ*hopM1*), or both (Δ*avrE1*Δ*hopM1*) paralleled that of ΔCEL+HopM1, ΔCEL+AvrE1, and *Pto* ΔCEL, respectively (S9 Fig).

To confirm that the observed phenotypes resulted from disruption of the B’ *α* and *β* genes, we constructed transgenic plants that express native promoter driven B’-GFP constructs, or 35S promoter driven GFP-B’ constructs. The growth defects of ΔCEL+AvrE1 and ΔCEL+HopM1 were restored in the homozygous T3 lines (Fig 5A) except for *NP*::*α-GFP* ln.19-3 and *35S*::*GFP-α* ln.1-6, which have ~20-fold higher or significantly lower levels of transcript than Col-0, respectively (Fig 5A and S8 Fig). Thus, for both B’ subunit mutants, complementation in multiple, independent plant lines restores the ability of AvrE1 and HopM1 to promote the growth of *Pto* ΔCEL.

The ability of AvrE1 and HopM1 to promote bacterial growth often correlates with their ability to suppress callose-containing cell wall fortifications. Analysis of callose deposition in our mutant collection revealed contributions of specific PP2A subunits to this host defense response and to the ability of AvrE1 to suppress it. The A subunit mutants did not affect the callose-suppressing function of either AvrE1 or HopM1. However, the *rcn1-6* mutant showed reduced callose deposition induced by the *Pto* ΔCEL mutant strain (Fig 5B). Notably, AvrE1 required B’α subunit, specifically, to fully suppress *Pto*-induced callose deposition and suppression of callose deposition by this T3E was restored in the complemented lines (Fig 5B and S9 Fig). The dependence on B’α for suppression of callose by AvrE1, but not HopM1, indicates that signaling dependent on a specific PP2A subunit differentiates one of the seemingly convergent functions of these T3Es. Notably, the callose phenotype does not fully parallel the bacterial growth phenotype. Callose deposition is a read-out for cell wall fortification, which is just one facet of the overall defense response responsible for limiting bacterial growth. The PP2A B’ subunits likely contribute to other aspects of plant defense in addition to callose deposition.

The observation that HopM1 requires AvrE1-associating PP2A B’ subunits for its growth promoting activity prompted us to test for association between HopM1 and these proteins. Dexamethasone (Dex)-inducible His-HopM1 [25] and selected GFP-tagged PP2A B’ subunit proteins were expressed by *Agro*-transient in *N*. *benthamiana*. HopM1 is known to induce proteasome-mediated degradation of the proteins with which it interacts, such as the AtMINs [25]. However, co-expression with His-HopM1 affected neither the apparent mobility nor abundance of PP2A B’ α, β, ι, or η proteins (S10A Fig). Also, co-immunoprecipitation of the His-tagged HopM1 protein was not detected with any of these PP2A B’ proteins (S10B Fig). Therefore, despite the requirement of PP2A B’α and β subunits for its virulence function, HopM1 is unlikely to interact directly with these phosphatase subunits. This led us to examine effects on the transcript levels of PP2A B’ subunits, which we already knew were induced dependent on delivery of T3Es by *Pto* DC3000 (Fig 3B). Notably, *Pto* ΔCEL failed to induce the transcripts of B’*α* and *β* subunits and induction of these transcripts was restored when ΔCEL expressed HopM1 (S10C Fig). Thus, unlike AvrE1 that apparently targets PP2A via association with B’α and β subunits, HopM1 may target PP2A indirectly by increasing the expression of genes encoding PP2A subunits, including *B’α* and *β*. However, the over-expression of PP2A B’α and β in complementation lines did not phenocopy HopM1 activity, *i*.*e*. did not restore the growth of *Pto* ΔCEL. Possible explanations include differences between HopM1-mediated induction of subunit expression and steady state over-expression, and limitation of activity when only a single subunit is over-expressed. Finally, given that ability of HopM1 to suppress both salicylic acid (SA)-dependent and SA-independent defense responses in Arabidopsis [13, 52, 53], as well as the ability of AvrE1, WtsE, and DspA/E to modulate SA accumulation or signaling in their respective host plants [8, 13, 54, 55], we tested the effect of SA on expression of the B’ subunits. However, unlike the known SA-responsive gene, *PR-1*, the levels of none of the B’ transcripts were affected following SA treatment (S10D Fig).

Given our observations that two different AvrE-family T3Es target PP2A and the wide distribution of this T3E family among plant pathogenic bacteria [1], we speculated that PP2A would play an important role in plant defense against diverse bacteria. To test this hypothesis, we measured the growth of a DspA/E (an AvrE-family T3E) encoding bacterial pathogen *Pectobacterium carotovorum* subsp. *carotovorum* (*Pcc*), which causes potato soft rot and can infect a wide range of plants, including Arabidopsis, under favorable environmental conditions. Following infiltration of wild-type *Pcc* into Arabidopsis leaves, we observed that each line in our collection of PP2A A and B’ subunit mutants, except for *b’γ*, supported higher growth of *Pcc* (S9C Fig). Because it is not known if virulence of *Pcc* depends on DspA/E in this assay, the relationship of DspA/E to PP2A is unclear. However, it is apparent that PP2A function in general, and specific B’ subunit-containing isoforms in particular, contribute positively to the ability of Arabidopsis to defend itself against this predominantly necrotrophic pathogen.

### AvrE1-associating PP2A B’ subunits modulate flg22-induced ROS production

**Fig 6:**
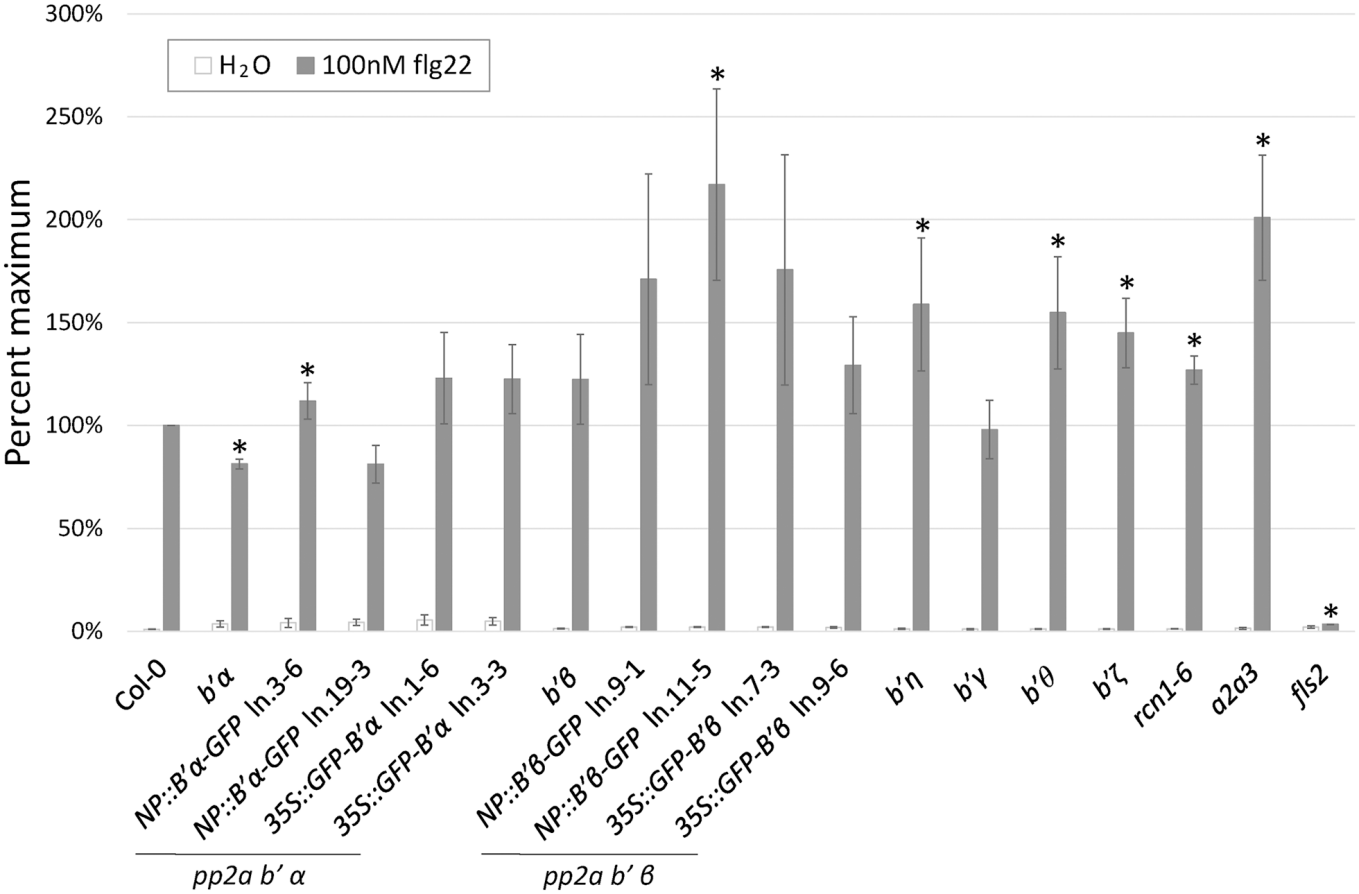
Specific PP2A isoforms modulate early flg22 signaling. Specific PP2A subunits differentially regulate flg22-induced reactive oxygen species (ROS) production. Leaf discs (6 mm in diameter) from 5-week-old plants of indicated genotypes were treated with 100nM flg22 or H2O and ROS production was monitored over-time. Data was normalized within each biological replicate with the average of highest relative light unit (RLU) value and the values preceding and following the peak value from flg22 treatment of Col-0 set to 100%. Shown are the averages of the normalized values of both the peak and the normalized values of the measurements preceding and following the peak from 3–6 biological replicates. * indicates a significant difference by Student’s t-test at P<0.05 comparing each genotype to Col-0.

PP2A complexes, specifically isoforms containing A1, B’ η or ζ, and C4 subunits, recently were shown to regulate the phosphorylation status of BAK1 and therefore attenuate the flg22/elf18-induced early PAMP signaling [40]. B’α and β were not tested in this study, but given their direct and indirect targeting by AvrE1 and HopM1, respectively, as well as the broader role of PP2A in controlling *Pcc* growth, we speculated that diverse PP2A subunits might regulate PAMP-triggered immunity (PTI, plant defenses triggered upon PAMP perception). To test this hypothesis we measured flg22 (22 amino acid PAMP from within bacterial flagellin)-induced reactive oxygen species (ROS) production, which is an early readout of PTI, in our collection of Arabidopsis PP2A A and B’ subunit mutants (Fig 6). Consistent with Segonzac et al. [40], higher ROS production was observed in the *rcn1-6*, *b’η* and *b’ζ* mutants. Additionally, *a2a3* and *b’ϑ* also showed higher ROS production suggesting that they may also negatively regulate early flg22 signaling. Notably, *b’α* mutant showed reduced ROS production compared to Col-0, and ROS production was restored in the complementation lines. Although ROS production was not affected in the *b’β* mutant tested, complementation lines that over-express *B’β* transcript showed elevated ROS production (Fig 6 and S11A Fig). Elevated ROS production in the *rcn1-6* and *b’η* mutants may be attributable to higher FLS2 (flagellin-sensitive 2, the Arabidopsis LRR-RLK receptor of flg22) protein levels (S11B Fig). However, most of the mutants affected ROS production without affecting steady-state FLS2 protein levels, indicating the important role of PP2A regulatory subunits in early FLS2 signaling. The contrasting phenotypes observed in mutants of the AvrE1-associating and non-associating B’ subunits indicates that AvrE1 may differentially target specific isoforms of PP2A that positively regulate early PTI signaling, through association with their B’ subunits. AvrE1 may disrupt the normal function of PP2A by disrupting binding to proper substrates or by redirecting PP2A to different cellular compartments.

## Discussion

AvrE-family T3Es are widely distributed among plant-pathogenic bacteria where they contribute to the formation of water-soaked lesions that are perhaps the most common symptom of bacterial diseases. Water-soaking likely releases nutrients from host cells to promote pathogen growth. AvrE-family T3Es also suppress host defenses, perhaps to limit defense induction resulting from the symptoms they induce. Thus, understanding the mode of action of AvrE-family T3Es is an essential step towards combating the many pathogens that deploy them. We employed two independent screening approaches, Y2H and SGA, that together highlighted PP2A B’ subunits as WtsE-interacting proteins and potential virulence targets. That PP2A is required for the virulence activity of WtsE *in planta* was supported by the observation that co-infiltration of *Pnss* with cantharidin, an inhibitor of PP2A, blocked WtsE-induced cell death in maize and perturbation of aromatic amino acid metabolism in a dose-dependent manner. Similar to its effect on WtsE, cantharidin also inhibited the cell death inducing activity of AvrE1 from *Pto* DC3000 in *N*. *benthamiana*. AvrE1 associated *in planta* with WIP1 and WIP2 as well as their closest Arabidopsis homologues. Moreover, both AvrE1 and the functionally redundant effector, HopM1, require the Arabidopsis PP2A B’ proteins for their virulence functions. However, HopM1 appears to target PP2A indirectly, perhaps via altered expression of B’ subunits. Within our working model for the role of PP2A-targeting T3Es on plant immune responses (S12 Fig), the direct effect(s) on PP2A may include: 1) altering phosphatase activity, 2) disrupting binding to normal substrates, and/or 3) driving interaction with novel substrates, perhaps through altering subcellular localization.

### AvrE-family T3Es share conserved virulence targets

The hypothesis that AvrE-family T3Es share conserved virulence target(s) across plant species has been raised previously based on the reports that AvrE1 could partially complement the virulence of the *dspA/E* mutant strain of *E*. *amylovora* [23], and that DspA/E was able to cross-complement the virulence of a *wtsE* mutant strain of *Pnss* [10], as well as the presence of conserved functional motifs in the effector proteins [21, 24]. This hypothesis is further strengthened by our observation that WtsE can complement the virulence of the *Pto* ΔCEL mutant strain in a manner similar to that of AvrE1 (S2 Fig). Moreover, AvrE1 associates not only with the Arabidopsis PP2A B’ subunit protein, but also with the maize WtsE-interacting proteins, WIP1 and WIP2 (Fig 4D). Therefore, we propose that PP2A B’ subunits are a conserved virulence target of the AvrE-family T3Es.

Our results and those of Siamer et. al. (2014) indicate that AvrE-family effector proteins also target a similar cellular pathway in yeast. Yeast suppressors of DspA/E toxicity included mutants of genes encoding two PP2A subunits, cdc55 (pp2a b) and ppm1 (protein carboxyl methyltransferase that methylates the C-terminus of PP2A catalytic subunit). ppm1 was also identified in the SGA screen for WtsE suppressors along with three other PP2A-related genes, including rts1 (pp2a b’). It is remarkable that the ability of DspA/E and WtsE to arrest yeast cell growth depends on two sequence unrelated PP2A B subunits, CDC55, a B regulatory subunit, and RTS1, a B’ regulatory subunit, respectively [46]. Based on our results, we posit that WtsE interacts directly with RTS1. Whether DspA/E interacts directly with CDC55 is unclear. Furthermore, growth of yeast expressing DspA/E also was restored by mutations that disrupt the ORM1/2 proteins, which negatively regulate serine palmitoyltransferase (SPT) that carries out the first and rate-limiting step of sphingolipid biosynthesis. PP2A heterotrimers containing CDC55 dephosphorylate, and thus activate, the ORM1/2 proteins [46, 56]. Mutations in ORM1/2 were not identified in our SGA screen with WtsE, perhaps because WtsE perturbs yeast by a distinct mechanism involving the targeting of RTS1-containing PP2A isoforms. Despite their differences in targeting of yeast PP2A sub-components, both WtsE and DspA/E perturb yeast sphingolipid biosynthesis, as evidenced by the restored growth of yeast expressing each effector by LCBs (S1B Fig) and many of the same yeast mutations that disrupt ceramide synthesis (Table 2). Although AvrE-family effectors may have evolved to target specific PP2A isoforms to carry out their virulence functions in different host plants, their actions in yeast, with its simpler repertoire of PP2A subunits, collapse onto the same sphingolipid biosynthetic pathway.

Another common target of AvrE-family effectors are LRR-RLKs. Similar to our finding that WtsE-N’ interacts with three maize LRR-RLKs, a Y2H screen identified four apple LRR-RLKs that interact with the N-terminal half of DspA/E from *Erwinia amylovora* [41]. Moreover, the maize LRR-RLKs (WIP3, 4, and 5) share moderate amino acid sequence similarities with the apple LRR-RLKs (DIPMs) ranging from 34% to 59% (Table 1). Although the significance of these interactions has not been demonstrated, the role of LRR-RLKs in activating plant defense responses inhibited by AvrE-family T3Es, such as cell wall reinforcement, makes them a highly plausible target. It is tempting to speculate that, through interaction with both PP2A and LRR-RLKs, AvrE-family effectors juxtapose PP2A to suppress phospho-activation of the kinase domain of LRR-RLKs. Consistent with the hypothesized interaction of PP2A with LRR-RLKs, our data and data from Segonzac *et al*. [40] show that mutation of specific PP2A subunits leads to enhanced early PAMP-signaling.

It is worth noting that our Co-IP and BiFC results indicate that multiple domains of AvrE1 associate with plant PP2A B’ subunits, whereas Y2H detected interaction of maize PP2A B’ subunits with only the N-terminal half of WtsE. We hypothesize that multiple, non-overlapping domains of AvrE-family T3Es interact with PP2A B’ subunits, and our ability to detect those interactions is variable between the different protein combinations and assay systems.

### Potential significance of targeting PP2A

Mounting evidence indicates that the major serine/threonine phosphatase PP2A regulates plant immunity. Arabidopsis isoforms of PP2A containing A1, B’η/ζ, and C4 negatively regulate early PAMP-signaling via modulating BAK1 phosphorylation status. [40]. In addition to confirming these results, our data also demonstrate that mutation of B’ *α* decreased flg22-induced ROS production without affecting the steady-state FLS2 protein levels. Thus, PP2A isoforms containing the B’ *α* subunit positively contribute to early PAMP-signaling. Collectively, these studies highlight subunit specificity as a key determinant of the role of PP2A in plant defense. An example revealing the complexity of the system is the differential contribution of PP2A subunits to defense-associated cell wall reinforcement; the A1 subunit (RCN1), which accounts for more than half of the total PP2A activity in Arabidopsis [57], positively contributes to pathogen-induced cell wall reinforcement while specific B’ subunits are required for the ability of AvrE-family T3Es to negatively regulate cell wall reinforcement.

The effect of the A1 subunit on cell wall reinforcement may be explained by its involvement in ethylene biosynthesis and signaling. Ethylene production triggered upon pathogen attack, acts both positively and negatively on plant immunity[58], including a positive contribution to the PAMP-triggered callose deposition into cell wall reinforcements through the activation of indole glucosinolate (IG) metabolism [59]. Notably, AvrE-family T3Es inhibit IG-signaling-dependent cell wall reinforcement in Arabidopsis [14, 52]. PP2A affects Arabidopsis ethylene biosynthesis and signaling by regulating the type-I ACS enzymes and the signaling node CTR1, a Raf-like kinase [38, 60]. Although ethylene production is constitutively elevated in *rcn1* mutant plants, further induction of ethylene by flg22 was reduced, possibly due to the interruption of the normal feedback loop or the contribution of RCN1 to flg22 signaling. Thus, perturbed ethylene-signaling and IG metabolism may explain the observed reduction in callose deposition in the *rcn1-6* mutant plants (Fig 5) and this supports a hypothesis that AvrE1 and HopM1 engage PP2A to hijack the ethylene pathway.

It is also reasonable to speculate that AvrE-family T3Es may have evolved to mimic eukaryotic phosphatase substrates. A number of *P*. *syringae* T3Es have been shown to utilize host posttranslational machinery to achieve proper cellular compartmentalization and/or to achieve full virulence functions, such as AvrPtoB, AvrRpm1 and AvrB [61–63]. Although no functional phosphorylation sites on AvrE-family T3Es have been reported so far, we cannot rule out the possibility that modification of AvrE-family T3Es by plant PP2A proteins may be essential for their virulence functions.

The multiple isomers of each PP2A subunit allow the formation of numerous holoenzymes, each with specific functions and substrates. Current data indicate that various PP2A holoenzymes act as both activators and suppressors of plant immunity. Pathogens may have evolved effector proteins such as AvrE-family T3Es to differentially target specific PP2A isoforms to their advantage. To further elucidate the mode of action of these T3Es in virulence and the role of the targeted PP2A isoforms in host defense, it will be crucial to determine the defense sectors regulated by these T3Es via their targeting of specific PP2A isoforms. It will also be of interest to determine if AvrE-family T3Es interact with specific B’ subunits to modulate PP2A phosphatase activity, subcellular localization, substrate specificity, and/or to serve as a direct substrate of PP2A.

## Materials and Methods

### Plants and growth conditions

*Arabidopsis thaliana* plants used in this work were of the Col-0 ecotype. Arabidopsis, and *Nicotiana benthamiana* plants were grown on Metro Mix 360 (Sun Gro Horticulture) at 22°C/16°C under an 8 h light (115 μmolm^-2^s^-1^)/16 h dark cycle. Sweet corn seedlings (*Zea mays* cv. Seneca Horizon) were grown and maintained in growth chambers set to 30°C, and a cycle of 18 h light and 6 h dark. The following Arabidopsis T-DNA insertion mutant lines were used: SALK_059903 (*rcn1-6*) [64], SALK_042724, SALK_014113 (*a2a3* double mutant) [65], SALK_077700 (*pp2ab’α*), SALK_151740C (*pp2ab’β*) (Arabidopsis Biological Resource Center), SALK_039172 (*pp2ab’ϒ*), SALK_057440 (*pp2ab’η*), SALK_107944c (*pp2ab’ζ*), SAIL_300_B01 (*pp2ab’ϑ*) [66]. The primers used for genotyping and transcript confirmation are listed in S2 Table.

### Bacteria

*Pseudomonas syringae* pv. tomato DC3000 (*Pto* DC3000) and mutants were grown on King’s B (KB) plates containing appropriate antibiotics at 28°C. *Pto* DC3000 mutants used in this study are ΔCEL (CUCPB5115, [15]), ΔCEL+AvrE1 [25], ΔCEL+HopM1 [13], ΔCEL+WtsE (S1 Text), *Pto hrcC-* [67], Δ*avrE1*Δ*hopM1*, Δ*avrE1*, and Δ*hopM1* [11]. *Pantoea stewartii* subsp. *stewartii* (*Pnss*) and mutants were grown on LB plates containing appropriate antibiotics at 28°C. *Pnss* mutants used in this study are *Pnss wtsE* mutant (strain DM5101, miniTn5gus insertion at amino acid 43 in WtsE, [10]), and *Pnss hrpJ* mutant [20]. *Pectobacterium carotovorum* subsp. *carotovorum* were grown on LB plates containing appropriate antibiotics at 28°C. *Agrobacterium tumefaciens* GV3101 was used in this study and is routinely cultured on LB plates containing appropriate antibiotics at 28°C.

### Yeast-two-hybrid screen

WtsE-N’ (aa 1–964) or WtsE-C’ (aa 964–1835) fused to the GAL4 DNA-binding domain in the pAS1 vector [68] were screened against a maize cDNA library in pAD-GAL4-2.1 vector (Agilent technologies), which was derived from young maize seedlings (provided by Dr. Erich Grotewold, The Ohio State University, Columbus, Ohio) in the yeast strain PJ69-4a [69]. Positive colonies were selected on synthetic media lacking leucine and tryptophan (for selection of the prey and bait plasmids, respectively) and histidine (for the selection of interacting partners). Plasmid DNA was isolated from putative positives, and sequenced. cDNA fragments were subsequently introduced into pGADT7 and pGBKT7 (Clontech Laboratories) vectors, and re-transformed into PJ69-4a with the corresponding WtsE fragment constructs to confirm the screen results.

### Synthetic genetic array

The synthetic genetic array was performed as described [43, 70]. Briefly, the chromosomal *trp1* locus in yeast strain y7092 was replaced with full-length *wtsE* under the *GAL1* promoter to produce the starting strain. The *gus* gene under the *GAL1* promoter was independently integrated into the same *trp1* locus and used as a control strain. The starting strain and the control strain were then individually crossed to an ordered array of ~5000 viable gene deletion mutants (representing ~80% of all yeast genes) such that meiotic progeny harboring both mutations could be scored for fitness. The screening was performed three times with four replicates in each array. A computer-based scoring system was used to generate an estimate of the relative growth from the pixel areas of individual colonies, as measured from digital images of the double-mutant plates and comparing that to the control plates. Colony size was normalized by dividing each colony size by the mean colony size across the particular plate from which the colony was derived. Visual inspection was also performed as a confirmation of the automated scoring.

To confirm the requirement of RTS1 for WtsE-induced growth inhibition in yeast, the haploid SGA parent strain (y7092) was modified by transforming a pGAL-HO plasmid to promote mating type switch, then counter-selected against the plasmid [71]. The haploid SGA starting strain, containing a chromosomal copy of *wtsE* under control of a *GAL1* promoter, was modified by replacing wild-type chromosomal *RTS1* with an *rts1*-deletion fragment by targeted integration and confirmed by PCR. The two strains were crossed to produce isogenic haploid progeny that contained chromosomal copies of either wild-type *RTS1* or the *rts1*-deletion plus or minus a chromosomal copy of *wtsE*. Cultures were grown to saturation (~1 x 10^8^ cells/mL) in YP+4% glucose to repress expression of *wtsE* and 10-fold dilutions were spotted in 4 μL volumes onto YP+2% glucose and YP+2% galactose and incubated at 30°C for 4 days.

### Vacuum-infiltration of maize seedlings

*Pnss* strains were cultured overnight at 28°C in LB broth supplemented with appropriate antibiotics. Cultures were centrifuged at 2500 x g for 15 min, and resuspended in infiltration buffer (10 mM KPO4, pH 7.2, with 0.2% Tween40) so that the OD540 = 0.57 (10^9^ CFU/mL). Six-day old cv. Seneca Horizon sweet corn seedlings were used for infiltration. Pots were inverted over beakers so that the seedlings were submerged in inoculum or buffer that was supplemented with cantharidin as indicated. Seedlings were then vacuum-infiltrated three times, 5 min each, at a vacuum pressure of 200 mm Hg. Infiltrated seedlings were then placed in growth chambers with a humidity greater than 65%. At 20 hours after infiltration (hai), the first true leaf from each infiltrated plant was examined for disease symptom and 3.5–5.0 cm of the leaf tip was collected for electrolyte leakage or LC-MS/MS analysis.

### Electrolyte leakage assays

*Pnss*-inoculated sweet corn leaves were sampled at 20 hai. Six leaf tips were excised from the infiltrated area for each treatment and separated into three technical replicates. The excised leaves were scanned for later area calculation, placed into a 50 ml conical tube containing 30 mL of deionized water, and held at room temperature for 1 h on a reciprocal shaker at 50 strokes per min. After shaking, the conductivity of the fluid in each tube was measured using a WTW model Cond 330i conductivity meter with a TetraCon 325 probe (WTW, Weilheim, Germany). Leaf areas were calculated using ImageJ software (http://rsbweb.nih.gov/ij/), conductivity per cm^2^ of leaf area was calculated, and values for buffer infiltrated samples were subtracted from values for bacterial inoculated samples.

### Extraction and measurement of coumaroyl conjugates

Six-day old Senenca Horizon sweet corn seedlings were infiltrated with wild-type *Pnss* or the *Pnss wtsE* mutant supplemented with various concentrations of cantharidin. Leaf samples (100mg each) were collected at 20 hai, and ground in 800 μL 80% methanol. Formononetin (60 μM final concentration) was spiked into each sample as an internal standard. Insoluble debris was cleared by centrifugation at 25,000 x g for 10 min. The supernatant was analyzed by LC-MS/MS. Liquid chromatography was performed using a Polaris C18-A column (4.6 X 150 mm, 3 μm particle size; Agilent Technologies) at a flow rate of 300 μL/min. The mobile phases were A-water, and B-acetonitrile. 0.1% formic acid was added as an ion-pairing agent. Column linear gradient was 0 to 18 min, A = 85%; 18 to 32 min, A = 45%; 32 to 40 min, A = 0%; 40 to 44 min, A = 85%. Tandem mass spectrometry was performed using Varian 500-MS system in a positive ion electrospray mode. Coumaroyl-tyramine and coumaroyl-tryptamine were observed as mass-to-charge ratios of 284 and 307, respectively. For the two compounds, retention times as well as masses of product ions matched published values [8] and those of the pure synthetic compounds. Pure synthetic coumaroyl-tyramine and coumaroyl-tryptamine were serial diluted into a buffer-infiltrated, formononetin spiked sample to generate a standard curve. Statistical analysis on composite data from three biological replicates was carried out using one-way ANOVA followed by the Tukey HSD test (P<0.05) using SAS software (http://www.sas.com/en_us/home.html).

### Bacterial secretion assay

The WtsE induction and secretion assay was carried out as described previously [10] with minor modifications. Briefly, *Pnss* (carrying plasmid pRF205 that constitutively expresses the *HrpS* transcriptional enhancer), and a T3SS-deficient *hrpJ* mutant strain were grown overnight, resuspended in 40 ml of inducing medium (2 mM (NH4)2SO4, 1 mM MgSO4.7H20, 0.1% casamino acids, 100 mM MES, 1% sucrose, 1 mM K2HPO4, pH = 5.5) at OD600 = 0.3, and supplemented with the indicated concentrations of cantharidin. These cultures were grown at 22°C for 24 hours, until the OD600 was approximately 1.9–2.2 and then were centrifuged to collect the cells. The resulting pellet constituted the cellular fraction. The supernatant fraction was supplemented with 1 mM PMSF and proteins were precipitated with 10% trichloroacetic acid and pelleting by spinning at 12,000 x g for 30 min at 4°C. The protein samples were separated by electrophoresis in 8% SDS-polyacrylamide electrophoresis gels. WtsE was detected using antibodies to *E amylovora* DspA/E [23].

### Quantitative real-time PCR

Total RNA from leaves of five-week old Arabidopsis plants was isolated using the Plant RNeasy Mini Prep Kit (Qiagen) followed by DNase I (Invitrogen) treatment. RNA quality was determined by gel electrophoresis and quantitated using a nano-drop model ND-1000 (Thermo Scientific). cDNA was synthesized from 1 μg of RNA using Reverse Transcription System (Promega). Quantitative real-time PCR analyses were performed using a Bio-Rad iQ5 real-time PCR detection system with iQ SYBR green supermix (Bio-rad). a*ctin7* was used as internal control, and gene expression data was analyzed using iQ5 software (Bio-Rad). Statistical analysis on composite data from multiple biological replicates was carried out using one-way ANOVA followed by the Tukey HSD test (P<0.05) using SAS software. Primers used for real-time PCR are listed in S2 Table.

### *Agrobacterium*-mediated transient gene expression

*Agrobacterium tumefaciens* strains (GV3101) carrying expression constructs or empty vectors and a p19 expression plasmid were incubated overnight at 28°C with shaking in LB broth supplemented with appropriate antibiotics. Cultures were centrifuged, washed with infiltration buffer (10 mM MES, pH 5.6, 10 mM MgCl2, 0.5% glucose), and resuspended in infiltration buffer supplemented with 100 μM acetosyringone. The inocula were adjusted so that the final concentration for GFP-HopQ1-1 was OD600 = 1.0, all other expression constructs were OD600 = 0.5, and p19 was OD600 = 0.3. The inocula were then incubated with shaking at 28°C for at least 2 hours. For cantharidin co-infiltration, different concentrations of cantharidin (0, 0.3, 1, 3, or 5 μM) were added to the inoculum just prior to infiltration. The inocula were infiltrated into 5- to 6-week old *Nicotiana benthamiana* leaves from the abaxial side using a 1 mL needleless syringe. The plants were incubated under normal growth conditions for: 48 hours to allow the expression of proteins for co-immunoprecipitation or to determine the subcellular localization of GFP-tagged constructs using Nikon C90i confocal microscope; 3–5 days for BiFC, followed by visualization under Nikon eclipse 80i fluorescent microscope; or 24–72 hours for cantharidin co-infiltration assays. For the *Dex*::*His-HopM1* construct, infiltrated leaves were sprayed with 30 μM Dex in 0.002% Silwet-77 at 48 hai to induce HopM1 expression and samples were collected at 6 hours after spraying.

### Co-immunoprecipitation

HA-immunoprecipitation was carried out using an anti-HA immunoprecipitation kit (Sigma-Aldrich, IP0010) according to the manufacturer’s protocol. Briefly, 0.5 g tissue was ground in liquid nitrogen, homogenized in 600 μl lysis buffer [50 mM Tris-HCl, pH 7.5, 150 mM NaCl, 1% Nonidet P40, 0.5% sodium deoxycholate, 1 X protein inhibitor cocktail (Sigma-Aldrich), 5 mM DTT], and centrifuged for 10 min at 12,000 x g at 4°C. This supernatant was then combined with 15μl anti-HA agarose beads suspension and incubated for 3 hours at 4°C. Agarose beads were washed four times in 1x IP buffer, and one more time in 0.1x IP buffer. The washed agarose beads were resuspended in 30 μL loading buffer and heated to 95°C for 5 min. Beads were removed by centrifugation, and the flow-through was analyzed by SDS-PAGE. GFP-immunoprecipitation was carried out according to the protocol supplied with the Protein A-Agarose beads (Roche). Briefly, 0.5 g tissue was ground in liquid nitrogen with a mortar and pestle, homogenized in 1 mL lysis buffer [50 mM Tris-HCl, pH 7.5, 150 mM NaCl, 1% Nonidet P40, 0.5% sodium deoxycholate, 1 X protein inhibitor cocktail (Sigma-Aldrich), 5 mM DTT], and centrifuged for 10 min at 12,000 x g at 4°C. This supernatant was then combined with 50 μl protein A-agarose suspension (1:1 mix) and incubated for 3 hours at 4°C to pre-clear the sample. Anti-GFP polyclonal antibody (Abcam, ab6556) was added to the pre-cleared samples at a 1:1000 ratio and incubated for 1 hour at 4°C. Then 50 μl protein A-agarose suspension (1:1 mix) was added and incubated overnight at 4°C. Agarose beads were washed twice in lysis buffer, twice in washing buffer 1 (50 mM Tris-HCl, pH 7.5, 150 mM NaCl, 0.1% Nonidet P40, 0.05% sodium deoxycholate), and once in washing buffer 2 (50 mM Tris-HCl, pH 7.5, 0.1% Nonidet P40, 0.05% sodium deoxycholate). The washed agarose beads were resuspended in 30 μL loading buffer and heated to 95°C for 3 min. Beads were removed by centrifugation, and the supernatant was analyzed by SDS-PAGE. PP2A GFP-B’ proteins were detected using anti-GFP polyclonal antibody (Abcam, ab6556), AvrE1-HA fragments were detected using anti-HA monoclonal antibody (Roche, 3F10), and the His-HopM1 protein was detected with anti-His antibody (Sigma) at a dilution of 1:10,000 for all antibodies. Chemiluminescent detection and band quantification were done using the western ECL blotting substrate and ChemiDoc XTS system (Bio-Rad).

### Bacterial growth assays

*Pseudomonas syringae* or *Pectobacterium carotovorum* bacterial suspensions at OD600 = 0.0002 (10^5^ CFU/mL) in 10 mM MgCl2 were pressure-infiltrated with a needleless syringe into leaves of 5-week old Arabidopsis plants. The infiltrated plants were allowed to dry, and returned to normal growth conditions for 4 days. Nine leaf discs (8 mm diameter) per treatment were separated into three replicates with three discs per replicate and homogenized in 10 mM MgCl2 buffer. Bacterial titer was determined by plating serial dilutions and calculating CFU per cm^2^ of infiltrated leaf. Comparisons were made among bacterial strains within the same plant genotype. Statistical analysis on composite data from at least three biological replicates was carried out using one-way ANOVA followed by the Tukey HSD test (P<0.05) using SAS software.

### Callose staining and quantification

Callose staining of Arabidopsis leaves was carried out as previously described [72]. Briefly, leaves of five-week-old plants were infiltrated with various *Pto* strains (10^8^ CFU/mL) or buffer (10 mM MgCl2). Six leaves from two plants for each treatment were collected at 16 hai. Leaves were cleared with lactophenol (mixture of 1 volume of a 1: 1: 1: 1 volume mix of glycerol, saturated phenol, lactic acid, and deionized water and 2 volumes of 95% ethanol), washed with 50% ethanol and then with water. Callose was stained with 0.01% aniline blue in 150 mM K2HPO4 (pH 9.5). Stained leaves were mounted in 50% glycerol and visualized with a Nikon eclipse 80i microscope using a UV filter (340–380 nm excitation filter, 435–485 nm bandpass barrier filter). The number of callose deposits was counted using ImageJ software (http://rsbweb.nih.gov/ij/). Comparisons were made for the same bacterial strain across different plant genotypes. Statistical analysis on composite data from three to six biological replicates was carried out using a pair-wise t-test (P<0.05) using SAS software.

### Reactive oxygen species measurement

Leaf discs of 6 mm diameter were excised from leaves of 5-week old Arabidopsis using a cork borer and floated in 100 ul Milli-Q water in a 96-well plate (Costar; Fisher Scientific, 3912) with the adaxial side up. Each well contained one leaf disc and each treatment was set up with three technical replicates. The plate was incubated for 20 hrs under constant light and then water was removed and replaced with 100 ul of a solution containing 20 μM luminol (Sigma, A8500), 20 μg/ml horseradish peroxidase (Sigma, P6782), and water or 100 nM flg22 (95% pure from EZBiolab, Carmel, IN). Reactive oxygen species production was then measured over time using a plate reader (Berthold Technologies, Centro LB960). At least three biological replicates were performed for each plant genotype and data was normalized within each biological replicate. The average of the highest relative light unit (RLU) and the values preceding and following the peak value from the Col-0 flg22 treatment was set to 100%. Normalized peak value and the values preceding and following the peak value were averaged for each genotype and the averages from multiple biological replicates were pooled and analyzed by a pair-wise t-test comparing each genotype to Col-0.

### Accession numbers

*Arabidopsis thaliana* sequence data from this article can be found in the Arabidopsis Genome Initiative or GenBank/EMBL databases under the following accession numbers: PP2A A1/RCN1 (AT1G25490), PP2A A2 (AT3G25800), PP2A A3 (AT1G13320),PP2A B’α (AT5G03470), PP2A B’β (AT3G09880), PP2A B’ι (AT5G25510), PP2A B’ξ (AT3G54930), PP2A B’δ (AT3G26030), PP2A B’ϒ (AT4G15415), PP2A B’η (AT3G26020), PP2A B’ζ (AT3G21650), PP2A B’ϑ (AT1G13460), RIN4 (AT3G25070), *ACTIN7* (AT5G09810), and *PR-1* (AT2G14610). Maize sequence data from this article can be found in GenBank/EMBL databases under the following accession numbers: A). ESTs: WIP1 (AI621449), WIP2 (AW052973); other maize PP2A B’ subunits: BG320458, AI586835, AW165473, AW562969, and AW600513. B). Proteins: WIP1 (NP_001146864), WIP2 (NP_001148974), WIP3 (ACN25452), WIP4 (ACL53390), WIP5 (AFW89165); other maize PP2A B’ subunit proteins: NP_001169040, NP_001183245, NP_001146050, NP_001141673, NP_001105904, AFW77313, AFW74402. Bacterial effector protein sequence data can be found in the GenBank/EMBL databases under the following accession numbers: AvrE1 (NP_791204.1), WtsE (AAG01467.2), HopM1 (NP_791202.1), HopQ1-1 (NP_790716.1).

## Supporting Information

S1 Fig WtsE interacts with maize LRR-RLKs in the yeast-two-hybrid assay, and long chain base supplementation partially rescued yeast from WtsE-induced growth inhibition. (A) The N-terminal half of WtsE (WtsE-N’, aa 1–196) interacted with three maize LRR-RLK proteins (WIP3, WIP4, and WIP5). Interactions by the yeast-two hybrid assay are selected on synthetic drop-out media lacking leucine, tryptophan, and adenine for WIP3 or histidine (supplemented with 3-amino-1,2,4-triazole, 3AT) for WIP4 and WIP5. Pictures were taken at 48 h: results shown are representative of three biological replicates. (B) Supplementation of 15 μM of phytosphingosine (PHS) or dihydrosphingosine (DHS) partially restored growth of yeast expressing WtsE. Full-length WtsE expression is induced by galactose (2%), and suppressed by glucose (2%). Pictures were taken at 96 hours: results shown are representative of three biological replicates.
(TIF)

S2 Fig WtsE complements the virulence defect of the *Pto* ΔCEL mutant strain in Arabidopsis. (A-B) WtsE induces cell death in Arabidopsis leaves when expressed in *Pto* ΔCEL mutant strain. (A) *Pto* ΔCEL, ΔCEL+AvrE1, ΔCEL+WtsE strains were pressure-infiltrated into leaves of five-week-old Arabidopsis Col-0 plants at 10^8^ CFU/ml. Macroscopic tissue collapse was assessed at 24 hai. The number of leaves displayed cell death / the total number of leaves assessed are shown. (B) Electrolyte leakage of leaves treated as in (A) were measured from 4–28 hai. Graph shows the normalized values of electrolyte leakage after subtracting buffer infiltrated sample readings from all treatments. Shown is mean ± SD from 2 biological replicates for 4 and 8 h time points, and 3 biological replicates for time points 16–28 h. * indicate significant difference at P<0.05 by student’s t-test comparing ΔCEL with ΔCEL+WtsE at the same time point. (C) WtsE promotes *Pto* ΔCEL growth in Arabidopsis leaves. Bacterial growth in five-week-old Arabidopsis leaves was assayed at 4 days following infiltration of indicated strains at 10^5^ CFU/ml. Shown are mean ± SD from five biological replicates and data were analyzed by one-way ANOVA followed by student’s t-test. Different letters indicate significant difference at P<0.05. (D) WtsE suppresses callose deposition induced by *Pto* ΔCEL in Arabidopsis leaves. Callose deposition in five-week-old Arabidopsis leaves was assayed at 16 hours following infiltration of buffer (10 mM MgCl_2_) or indicated bacterial strains at 10^8^ CFU/ml. Shown are mean ± SEM from 4 biological replicates and data were analyzed by one-way ANOVA followed by Tukey test. Different letters indicate significant difference at P<0.05.
(TIFF)

S3 Fig Localization of Arabidopsis PP2A B’ subunit proteins and AvrE1 fragments in *N*. *benthamiana* leaf epidermal cells. (A) Arabidopsis PP2A B’ subunit proteins (α, β, ξ, ι, η) and AvrE1 fragments were fused to the C-terminus of GFP under the control of CaMV35S promoter and *Agro*-transiently expressed in *N*. *benthamiana* leaf epidermal cells together with PmCherry (plasma membrane marker). Confocal pictures were taken at 48 hai. Scale bars, 50 μm. (B) A microsomal fractionation assay was used to assess the subcellular distribution of the proteins expressed in *N*. *benthamiana* leaf epidermal cells (the tissue equivalents of S: M are ~1:20). Proteins in each fraction were detected by immunoblotting using anti-GFP or anti-HA antibodies. For both (A) and (B), YFP-RIN4 was used as a plasma membrane protein control and free GFP was used as a soluble protein control. C-terminal HA tagged AvrE1 fragment constructs showed similar subcellular distribution as the N-terminal GFP tagged constructs. Stars indicates predicted size of the corresponding full-length constructs.
(TIF)

S4 Fig Non-lethal fragments of AvrE1 associate with specific Arabidopsis PP2A B’ subunits in BiFC, and mutation of the AvrE1 putative C-terminal ERMRS motif does not affect its localization or association with PP2A B’ subunit proteins. (A) AvrE1 associates with specific PP2A B’ subunit proteins in bi-molecular fluorescence complementation (BiFC) assay. Both N and C-terminal AvrE1 fragments associate with PP2A B’α, β, and ι, but not with η. Shown is a representative result from three biological replicates. GFP^c^-AvrE1-M’, GFP^n^-AvrE1-M’, GFP^n^-ι, and GFP^c^-η are not shown due to auto-fluorescence. Scale bars, 50 μm. (B) The AvrE1-C’ fragment k1k2 mutant (KK1787-88AA, mutation within the ERMRS motif) does not affect association with PP2A B’α, β, and ι in *N*. *benthamiana* by co-immunoprecipitation. Shown is representative blot from two biological replicates. (C) AvrE1-C’ (k1k2) mutant showed similar distribution as the wild-type fragment in microsomal fractionation assays. Constructs were under the control of a CsVMV promoter and expressed for 48hrs in *N*. *benthamiana* epidermal cells by *Agro*-mediated transient expression. Arrows indicates predicted size of the corresponding full-length constructs.
(TIF)

S5 Fig Cantharidin attenuates AvrE1-induced cell death and facilitates AvrE1 expression in *N*. *Benthamiana*. Cantharidin at 0, 0.3, 1, 3, and 5 μM was co-infiltrated with *Agrobacterium* carrying either *35S*::*GFP-AvrE1* (full length) or *35S*::*GFP* empty vector at OD_600_ = 0.5. (A) Cantharidin inhibits *Agrobacterium*-mediated transient expression, which is apparent from reduced expression of free GFP. Nonetheless, cantharidin allows detectable expression of GFP fused to full-length AvrE1 prior to visible tissue collapse. Leaf pictures shown are taken at 48 hai representative of four biological replicates. Confocal microscopic pictures and western blots show protein expression at 24 hai representative of three biological replicates. Scale bars, 50 μm. (B) Microsomal fraction followed by GFP-immunoprecipitation shows full-length AvrE1 is only detectable in the microsomal fraction (tissue equivalent of S: M = 1: 1 in both input and IP fractions). Shown is representative blot from two biological replicates. (C) Cell death was quantified by measuring electrolyte leakage of the infiltrated samples. Graph shows the normalized values of electrolyte leakage after subtracting buffer infiltrated sample readings (no cantharidin) from GFP-AvrE1 or free GFP sample readings, then dividing by the total area of leaf discs. Graphs represent mean ± SEM from four biological replicates. (D) The graph shows the mean ± SEM from four biological replicates for the relative values of electrolyte leakage (GFP-AvrE1reading / total area—free GFPreading / total area). (E) Statistical analysis (ANOVA followed by t-test) on data acquired in (D) was performed at each time point comparing the effect of different concentrations of cantharidin on electrolyte leakage differences. Different letters indicate significant difference at that time point with P<0.05.
(TIFF)

S6 Fig Cantharidin accelerates HR-type cell death induced by HopQ1-1 in *N*. *Benthamiana*. Cantharidin at 0, 1, and 3 μM was co-infiltrated with *Agrobacterium* carrying either *35S*::*GFP-HopQ1-1* (0D_600_ = 1.0) or *35S*::*GFP* empty vector (0D_600_ = 0.5, supplemented with a filler strain expressing pCsVMV-HA3-N-1300 empty HA vector at 0D_600_ = 0.5). (A) Leaf pictures shown are taken at 70 hai. (B) Western blots show protein expression at 24 hai. Shown are representative images or blots from four independent biological replicates.
(TIF)

S7 Fig Association of full-length AvrE1 with Arabidopsis PP2A B’β is not detected by co-immunoprecipitation. *35S*::*GFP-AvrE1* was co-expressed with *35S*::*HA-B’β* in the presence of 1 μM cantharidin in *N*. *benthamiana* following *Agro*-transient expression. Co-immunoprecipitation of HA-B’β was tested using anti-HA antibody following GFP-pull down of full-length AvrE1. Shown is a representative blot from two biological replicates. Arrow indicates predicted size of full-length GFP-AvrE1 fusion protein.
(TIF)

S8 Fig Arabidopsis PP2A subunit mutants. (A) Schematics of PP2A T-DNA insertion mutants used in this study. A subunit mutants: *rcn1-6* and *a2a3*. B’ subunit mutants: *α*, *β*, *η*, *ϒ*, *ϑ*, and *ζ*. (B) Reverse transcription-PCR analysis of PP2A B’ subunit transcripts in different mutant backgrounds and complementation lines. cDNAs were generated from at least four independent plants of each genotype, and subjected to PCR or real-time PCR analysis using primers complementary to *α*, *β*, *ι*, *η*, *ϒ*, *ϑ*, *ζ*, and *actin7* transcripts.
(TIF)

S9 Fig Specific PP2A subunits differentially regulate Arabidopsis resistance to *Pto*DC3000 and *Pcc*. (A-B) *Pto* DC3000 mutant strains with deletions of AvrE1 and/or HopM1 exhibit comparable phenotypes to the corresponding plasmid complemented *Pto* ΔCEL strains. Similar to *Pto* ΔCEL, the double mutant *Pto* DC3000 strains, Δ*avrE1*Δ*hopM1*, grows less (A) and elicits more callose (B) than wild-type *Pto* DC3000 on wild-type Col-0 plants. Similarly, the single deletion mutant *Pto* DC3000 strains Δ*hopM1* and Δ*avrE1* also phenocopy ΔCEL+AvrE1 and ΔCEL+HopM1, respectively. (A) AvrE1 or HopM1, expressed in Δ*hopM1* or Δ*avrE1*, respectively, failed to promote bacterial growth in Arabidopsis *pp2a b’α* and *β* mutants, but retained function in *b’ η* mutant. Leaves of five-week-old Arabidopsis plants were infiltrated with indicated bacterial strains (10^5^ CFU/ml). Bacterial growth on different PP2A mutant plants was assayed four days after infiltration. Values shown are mean ± SEM from three biological replicates. Data was analyzed by one-way ANOVA followed by the Tukey test comparing different bacterial strains on individual plant genotypes. Different letters of the same style (eg. A vs B, or A’ vs B’) indicate a significant difference at P<0.05 within the same plant genotype. (B) AvrE1, expressed in Δ*hopM1* just as in ΔCEL+AvrE1, was unable to fully suppress callose in the Arabidopsis *pp2a b’ α* mutant. Leaves of five-week-old Arabidopsis plants were infiltrated with the indicated bacterial strains (10^8^ CFU/ml) or buffer (10 mM MgCl2), and collected at 16 hai for callose staining. Values shown are mean ± SEM from three biological replicates. ** indicates a significant difference by Student’s t-test at P<0.01. (C) Specific PP2A mutants are more susceptible to *Pectobacterium carotovorum* subsp. *carotovorum* (*Pcc*). Leaves of five-week-old Arabidopsis plants were infiltrated with wild-type *Pcc* (10^5^ CFU/ml). Bacterial growth on different PP2A mutant plants was assayed four days after infiltration. Values shown are mean ± SD from three biological replicates. * indicates a statistically significant difference at P<0.05 by Student’s t-test comparing growth in mutants to that in Col-0.
(TIF)

S10 Fig HopM1 engages PP2A B’ subunits by inducing gene expression rather than direct interaction. (A) HopM1 does not degrade PP2A B’ subunit proteins. At 48 hours following *Agrobacterium*-mediated transient expression in *N*. *benthamiana*, HopM1 expression was induced by spraying with 0.002% Silwet-77 with or without 30 μM Dex. At six hours after spraying, samples were collected for immunoblotting. (B) HopM1 does not detectably co-immunoprecipitate with PP2A B’ subunits. Samples prepared as in (A) were analyzed by co-immunoprecipitation, PP2A B’ subunit proteins were pulled-down with anti-GFP antibody and GFP-B’ subunits and His-HopM1 were detected by immunoblotting. Shown are representative blots from two biological replicates. (C) Transcripts of PP2A B’ *α* and *β* were induced by HopM1. Five-week-old Col-0 plants were infiltrated with indicated *Pto* strains at 10^8^ CFU/ml and infiltrated leaves were collected at nine hai. Values shown are mean ± SEM from five to six biological replicates. * indicates a statistically significant difference by Student’s t-test at P<0.05. (D) Transcription of Arabidopsis PP2A B’ subunit genes are not affected by SA. Five-week-old Col-0 Arabidopsis leaves were sprayed with 500 μM SA (with 0.0002% Silwet-77) or buffer (H2O + 0.1% ethanol + 0.0002% Silwet-77). Samples were collected at 6, 12, and 24 hai and subjected to qRT-PCR. Values shown are mean ± SEM of normalized data from three biological replicates with transcript level in untreated samples set to 1. *PP2A B’* genes are shown in regular scale, *PR-1* transcript level is shown in log10 scale. Log transformed *PR-1* transcript levels for untreated samples are 0, and are thus not shown on the graph. ** indicates a significant difference by Student’s t-test at P<0.01.
(TIFF)

S11 Fig Specific PP2A isoforms modulate flg22-induced reactive oxygen species (ROS) production. (A) The data shown was used to produce Fig 6. Leaf discs from five-week-old plants of the indicated genotypes were treated with 100 nM flg22 or H2O and ROS productions was monitored over time. Data was normalized within each biological replicate with the average value of the highest relative light unit (RLU) and the values preceding and following the peak value from the Col-0 flg22 treatment set to 100%. Shown are mean ± SEM from 3–6 biological replicates. (B). Steady-state FLS2 protein levels in PP2A mutants and complementation lines. Protein was extracted from untreated leaves from 4–6 individual plants of the same genotype and FLS2 protein was detected using polyclonal antibody against FLS2 [73]. Shown are representative blots from two biological replicates, numbers indicate average band intensity from both replicates with the band intensity of Col-0 set to 1.
(TIF)

S12 Fig Working model: AvrE-family T3Es modulate plant immunity via interaction with PP2A complexes. AvrE-family T3Es, including AvrE1 from *Pseudomonas syringae* pv. tomato, and WtsE from P*antoea stewartii* pv. *stewartii*, associate with B’ subunits of host protein phosphatase 2A complexes to exploit their phosphatase activity. Potential mechanisms for modulation of plant immunity include: a. inhibition of known PAMP-recognition receptor complexes such as the FLS2/BAK1 complex; b. inhibition of other plasma-membrane localized leucine-rich repeat receptor-like complexes (*e*.*g*. those of WIP3, 4, and 5 and DIMP1-4 [41]) whose role in plant immunity are yet to be determined; c. disruption of host sphignolipid homeostasis with possible effects on SA-signaling, polarized transportation of antimicrobial compounds, and host cell death; d. disruption of other defense sectors, possibly including ethylene signaling and/or transcriptional regulation of metabolic enzymes. HopM1 also requires specific host PP2A complexes to promote virulence, possibly via indirect transcriptional modulation. Solid lines indicate established effects, dashed lines with question marks indicate hypothesized effects.
(TIFF)

S1 Table Full synthetic genetic array (SGA) results. (XLS)

S2 Table Primers used in this study. (PDF)

S1 Text Supplemental Materials and Methods. (PDF)
