# Supplementary figures and images for: Direct and Indirect Targeting of PP2A by Conserved Bacterial Type-III Effector Proteins
Source: PLoS Pathog. 2016 May 18;12(5):e1005609. doi: 10.1371/journal.ppat.1005609 (PMC4871590; doi:10.1371/journal.ppat.1005609)

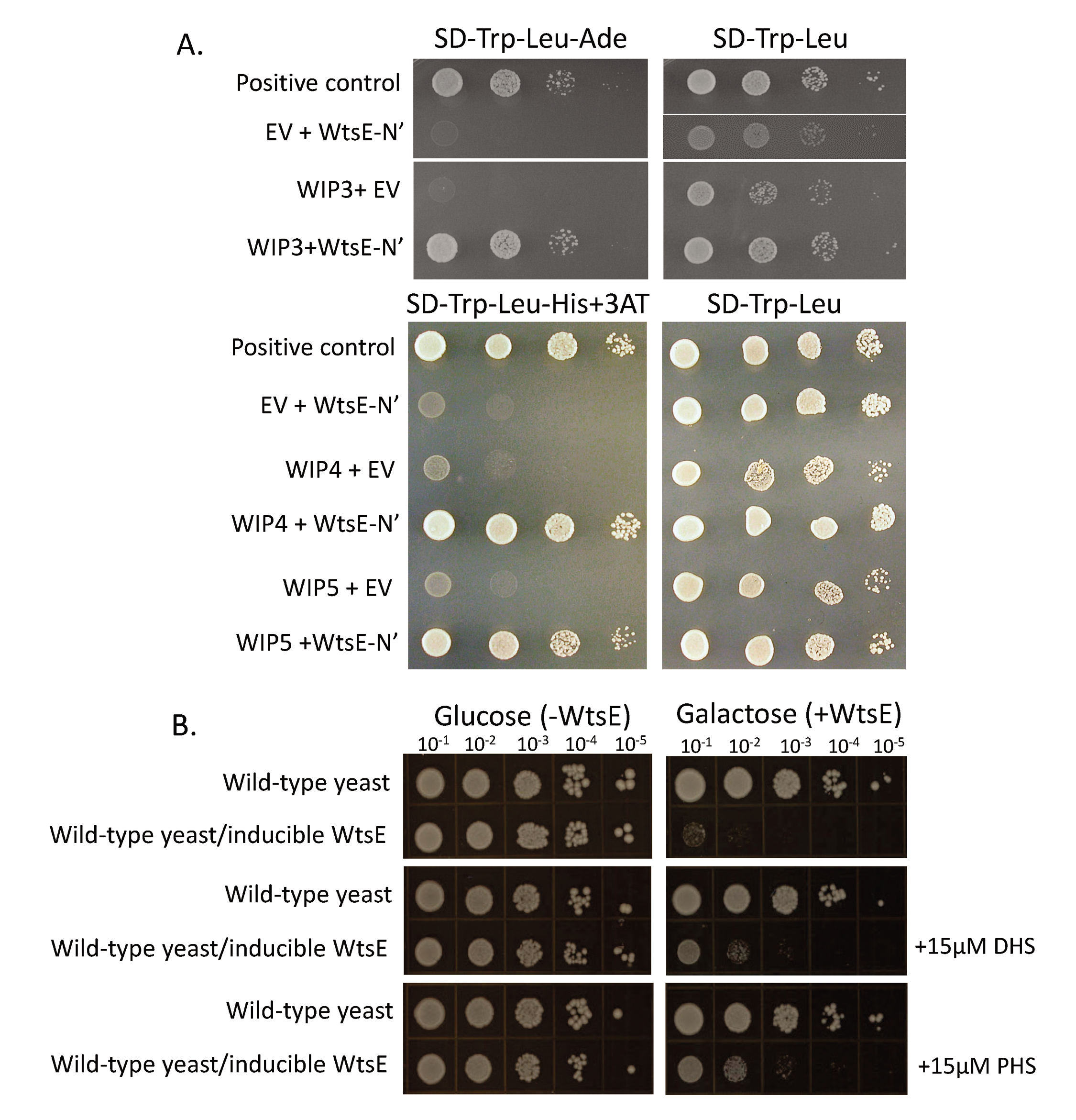

Supplement: S1 Fig — (A) The N-terminal half of WtsE (WtsE-N’, aa 1–196) interacted with three maize LRR-RLK proteins (WIP3, WIP4, and WIP5). Interactions by the yeast-two hybrid assay are selected on synthetic drop-out media lacking leucine, tryptophan, and adenine for WIP3 or histidine (supplemented with 3-amino-1,2,4-triazole, 3AT) for WIP4 and WIP5. Pictures were taken at 48 h: results shown are representative of three biological replicates. (B) Supplementation of 15 μM of phytosphingosine (PHS) or dihydrosphingosine (DHS) partially restored growth of yeast expressing WtsE. Full-length WtsE expression is induced by galactose (2%), and suppressed by glucose (2%). Pictures were taken at 96 hours: results shown are representative of three biological replicates. (TIF) [file ppat.1005609.s001.tif]

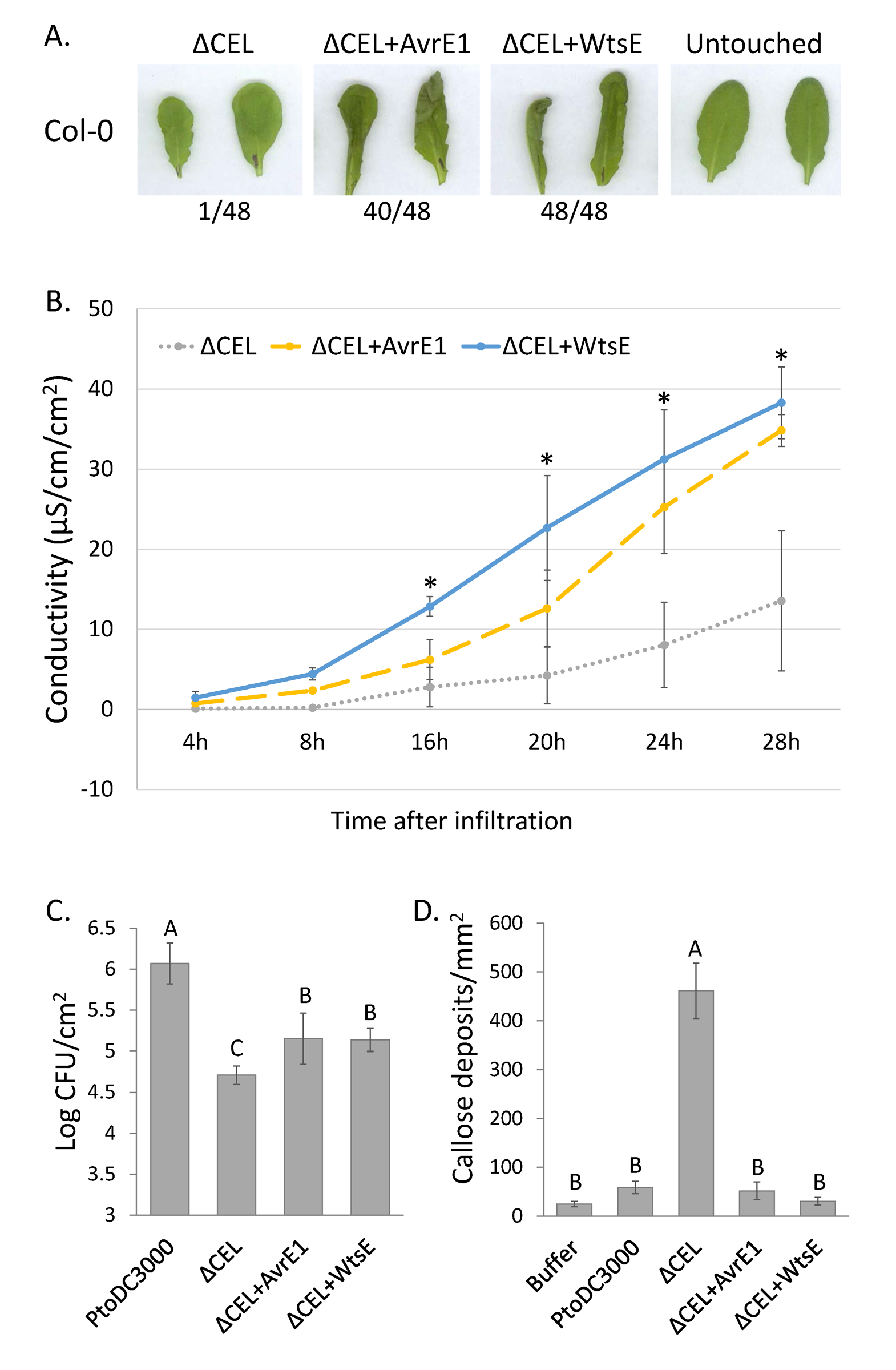

Supplement: S2 Fig — (A-B) WtsE induces cell death in Arabidopsis leaves when expressed in Pto ΔCEL mutant strain. (A) Pto ΔCEL, ΔCEL+AvrE1, ΔCEL+WtsE strains were pressure-infiltrated into leaves of five-week-old Arabidopsis Col-0 plants at 108 CFU/ml. Macroscopic tissue collapse was assessed at 24 hai. The number of leaves displayed cell death / the total number of leaves assessed are shown. (B) Electrolyte leakage of leaves treated as in (A) were measured from 4–28 hai. Graph shows the normalized values of electrolyte leakage after subtracting buffer infiltrated sample readings from all treatments. Shown is mean ± SD from 2 biological replicates for 4 and 8 h time points, and 3 biological replicates for time points 16–28 h. * indicate significant difference at P<0.05 by student’s t-test comparing ΔCEL with ΔCEL+WtsE at the same time point. (C) WtsE promotes Pto ΔCEL growth in Arabidopsis leaves. Bacterial growth in five-week-old Arabidopsis leaves was assayed at 4 days following infiltration of indicated strains at 105 CFU/ml. Shown are mean ± SD from five biological replicates and data were analyzed by one-way ANOVA followed by student’s t-test. Different letters indicate significant difference at P<0.05. (D) WtsE suppresses callose deposition induced by Pto ΔCEL in Arabidopsis leaves. Callose deposition in five-week-old Arabidopsis leaves was assayed at 16 hours following infiltration of buffer (10 mM MgCl2) or indicated bacterial strains at 108 CFU/ml. Shown are mean ± SEM from 4 biological replicates and data were analyzed by one-way ANOVA followed by Tukey test. Different letters indicate significant difference at P<0.05. (TIFF) [file ppat.1005609.s002.tiff]

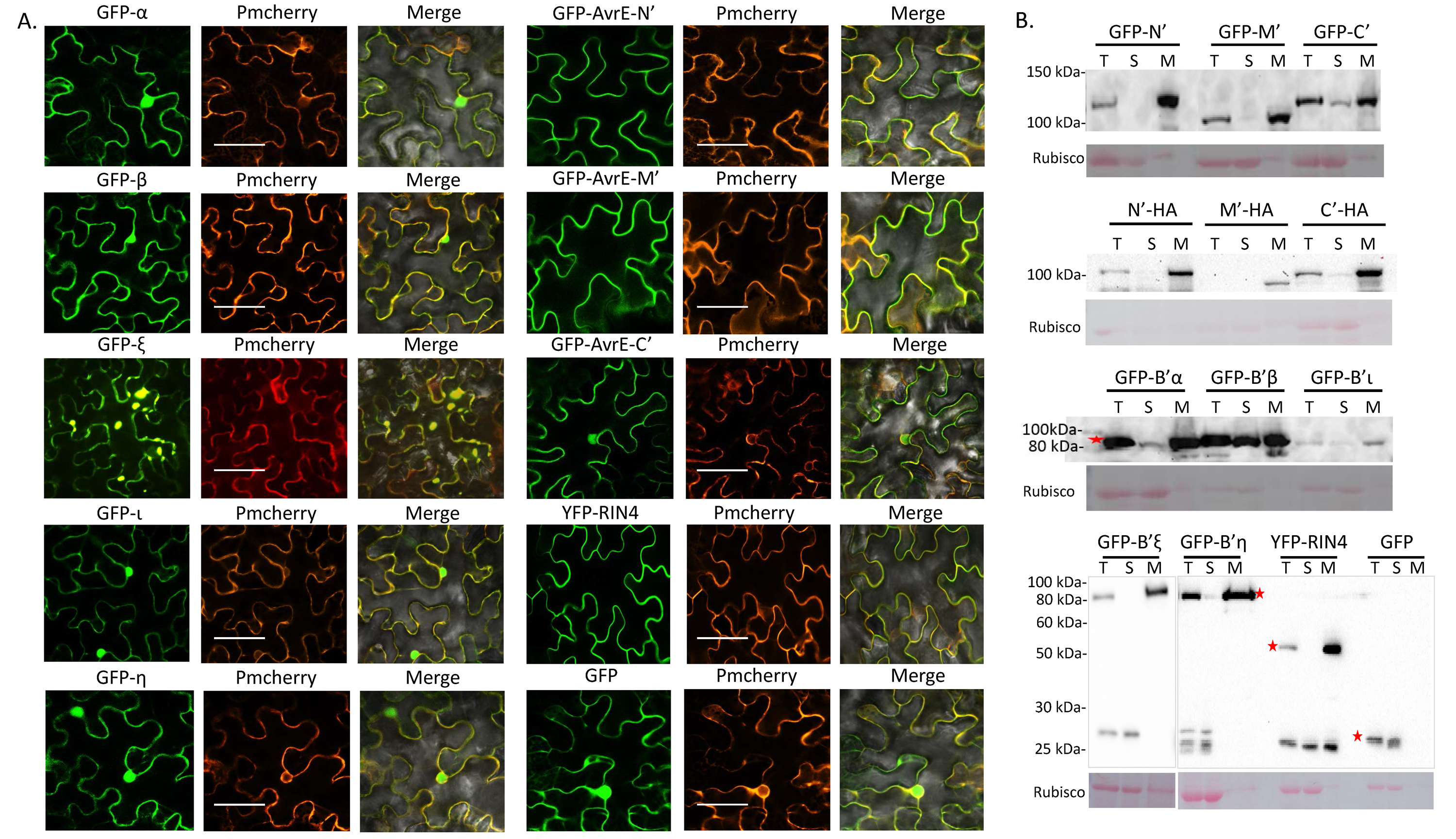

Supplement: S3 Fig — (A) Arabidopsis PP2A B’ subunit proteins (α, β, ξ, ι, η) and AvrE1 fragments were fused to the C-terminus of GFP under the control of CaMV35S promoter and Agro-transiently expressed in N. benthamiana leaf epidermal cells together with PmCherry (plasma membrane marker). Confocal pictures were taken at 48 hai. Scale bars, 50 μm. (B) A microsomal fractionation assay was used to assess the subcellular distribution of the proteins expressed in N. benthamiana leaf epidermal cells (the tissue equivalents of S: M are ~1:20). Proteins in each fraction were detected by immunoblotting using anti-GFP or anti-HA antibodies. For both (A) and (B), YFP-RIN4 was used as a plasma membrane protein control and free GFP was used as a soluble protein control. C-terminal HA tagged AvrE1 fragment constructs showed similar subcellular distribution as the N-terminal GFP tagged constructs. Stars indicates predicted size of the corresponding full-length constructs. (TIF) [file ppat.1005609.s003.tif]

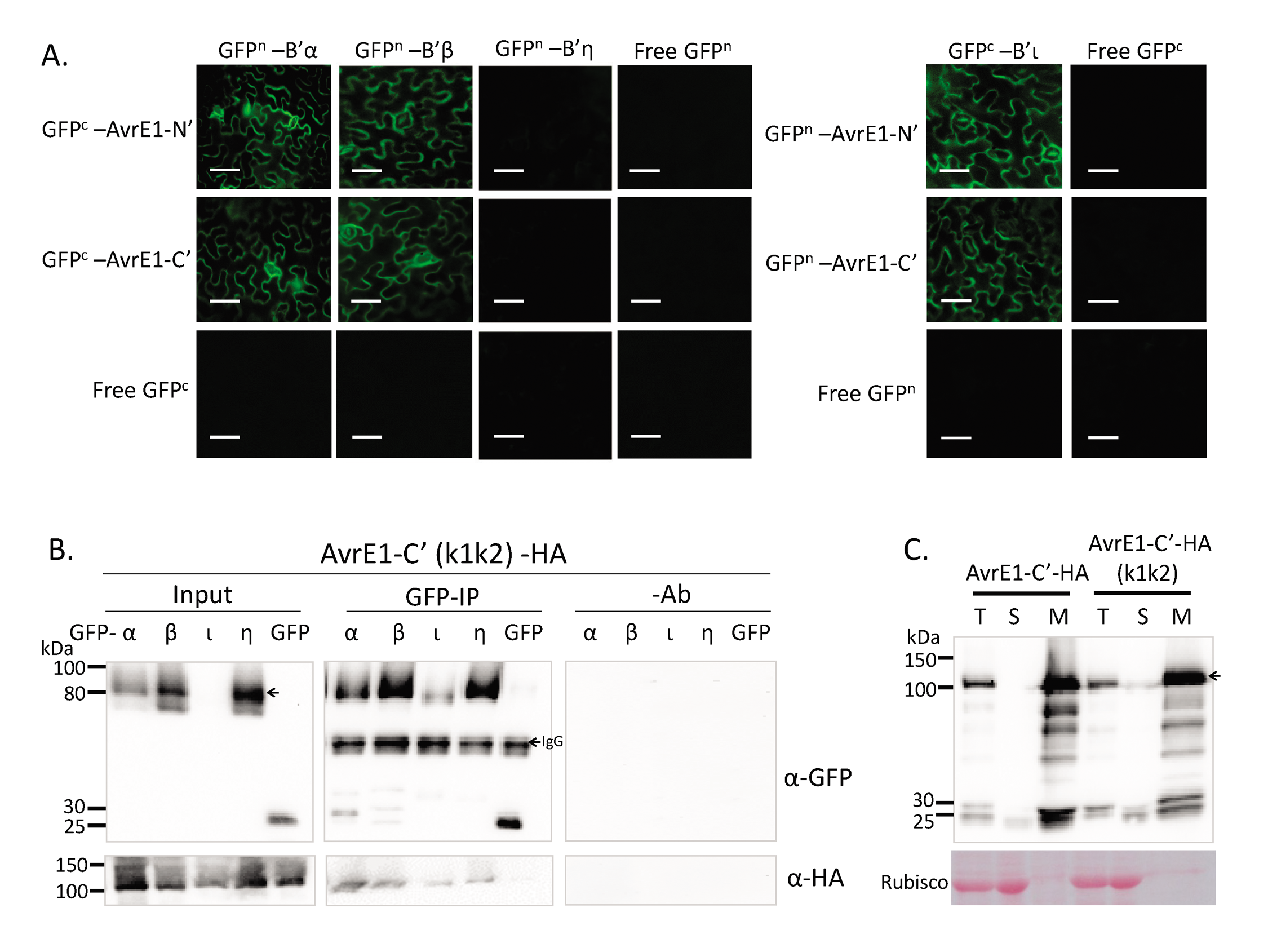

Supplement: S4 Fig — (A) AvrE1 associates with specific PP2A B’ subunit proteins in bi-molecular fluorescence complementation (BiFC) assay. Both N and C-terminal AvrE1 fragments associate with PP2A B’α, β, and ι, but not with η. Shown is a representative result from three biological replicates. GFPc-AvrE1-M’, GFPn-AvrE1-M’, GFPn-ι, and GFPc-η are not shown due to auto-fluorescence. Scale bars, 50 μm. (B) The AvrE1-C’ fragment k1k2 mutant (KK1787-88AA, mutation within the ERMRS motif) does not affect association with PP2A B’α, β, and ι in N. benthamiana by co-immunoprecipitation. Shown is representative blot from two biological replicates. (C) AvrE1-C’ (k1k2) mutant showed similar distribution as the wild-type fragment in microsomal fractionation assays. Constructs were under the control of a CsVMV promoter and expressed for 48hrs in N. benthamiana epidermal cells by Agro-mediated transient expression. Arrows indicates predicted size of the corresponding full-length constructs. (TIF) [file ppat.1005609.s004.tif]

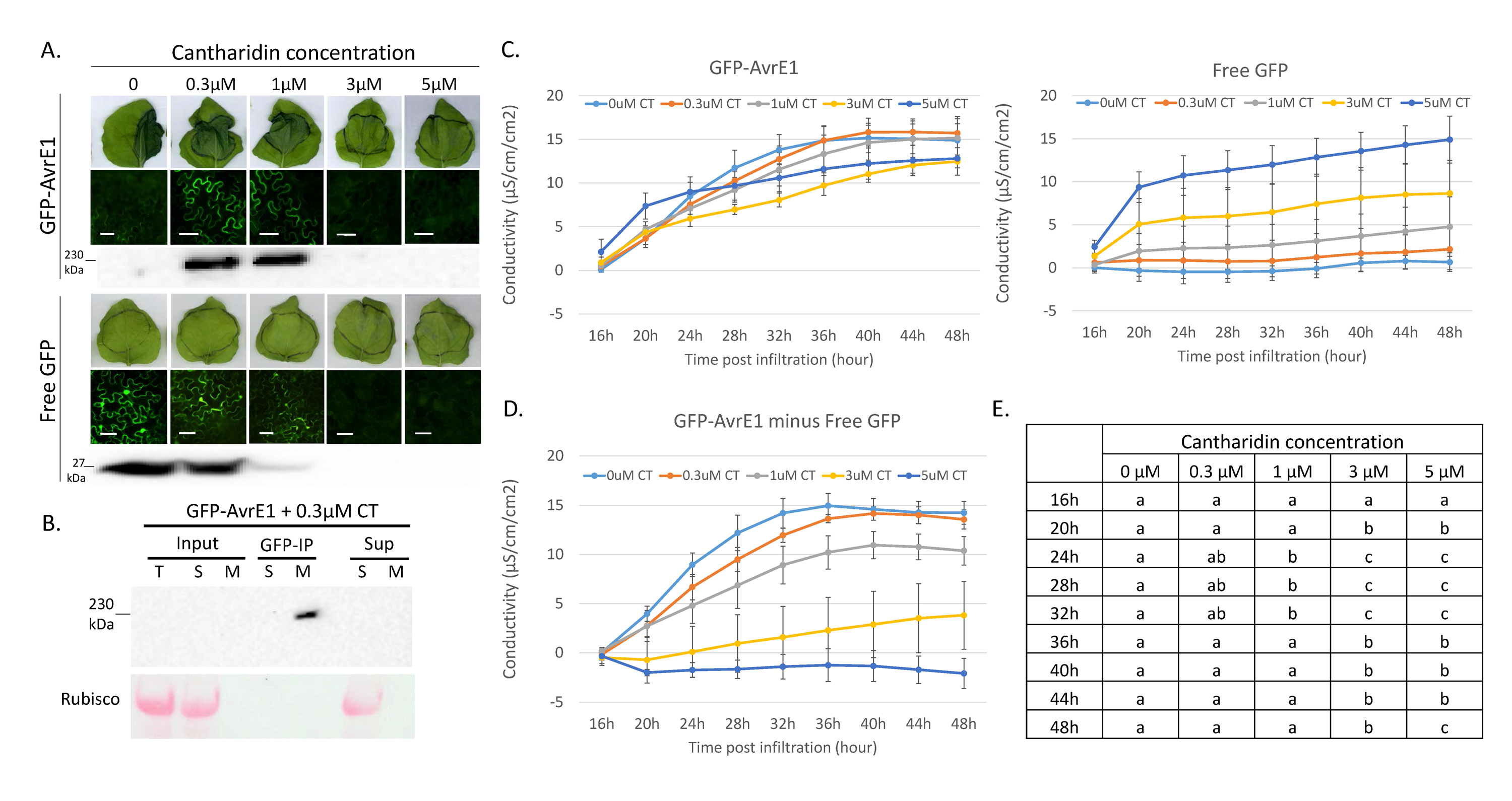

Supplement: S5 Fig — Cantharidin at 0, 0.3, 1, 3, and 5 μM was co-infiltrated with Agrobacterium carrying either 35S::GFP-AvrE1 (full length) or 35S::GFP empty vector at OD600 = 0.5. (A) Cantharidin inhibits Agrobacterium-mediated transient expression, which is apparent from reduced expression of free GFP. Nonetheless, cantharidin allows detectable expression of GFP fused to full-length AvrE1 prior to visible tissue collapse. Leaf pictures shown are taken at 48 hai representative of four biological replicates. Confocal microscopic pictures and western blots show protein expression at 24 hai representative of three biological replicates. Scale bars, 50 μm. (B) Microsomal fraction followed by GFP-immunoprecipitation shows full-length AvrE1 is only detectable in the microsomal fraction (tissue equivalent of S: M = 1: 1 in both input and IP fractions). Shown is representative blot from two biological replicates. (C) Cell death was quantified by measuring electrolyte leakage of the infiltrated samples. Graph shows the normalized values of electrolyte leakage after subtracting buffer infiltrated sample readings (no cantharidin) from GFP-AvrE1 or free GFP sample readings, then dividing by the total area of leaf discs. Graphs represent mean ± SEM from four biological replicates. (D) The graph shows the mean ± SEM from four biological replicates for the relative values of electrolyte leakage (GFP-AvrE1reading / total area—free GFPreading / total area). (E) Statistical analysis (ANOVA followed by t-test) on data acquired in (D) was performed at each time point comparing the effect of different concentrations of cantharidin on electrolyte leakage differences. Different letters indicate significant difference at that time point with P<0.05. (TIFF) [file ppat.1005609.s005.tiff]

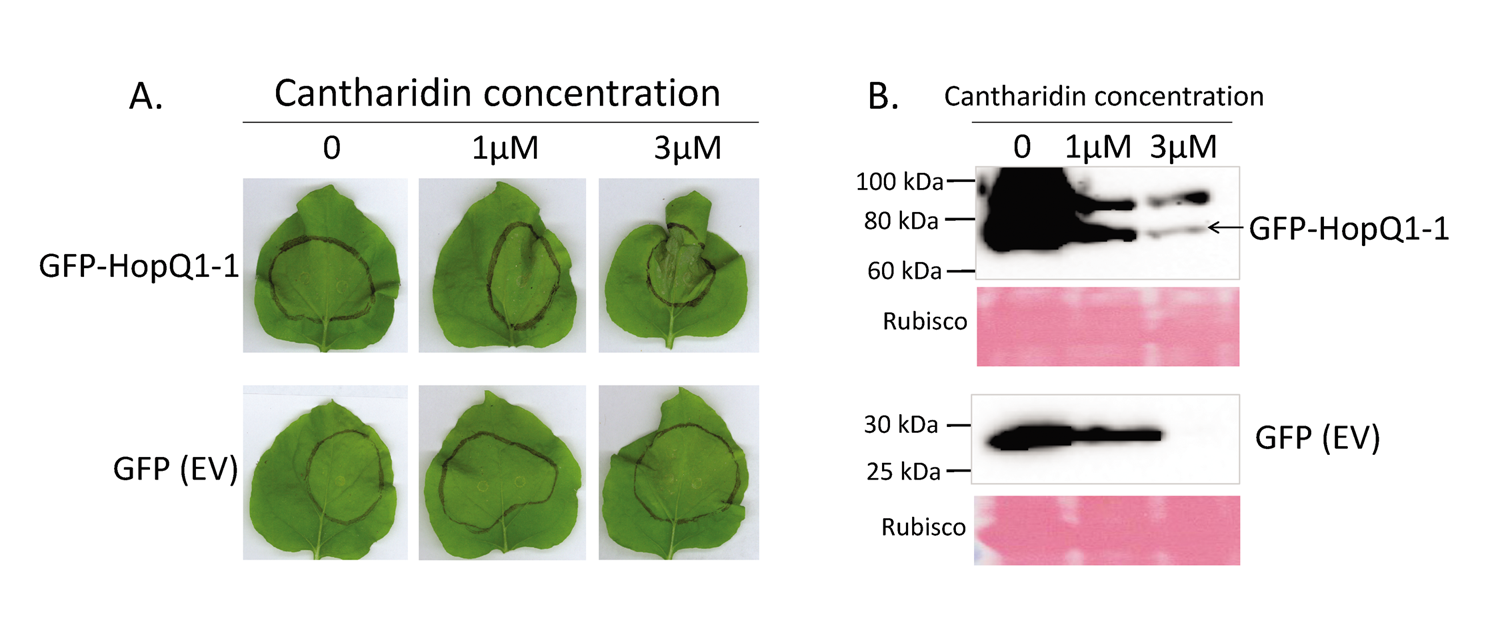

Supplement: S6 Fig — Cantharidin at 0, 1, and 3 μM was co-infiltrated with Agrobacterium carrying either 35S::GFP-HopQ1-1 (0D600 = 1.0) or 35S::GFP empty vector (0D600 = 0.5, supplemented with a filler strain expressing pCsVMV-HA3-N-1300 empty HA vector at 0D600 = 0.5). (A) Leaf pictures shown are taken at 70 hai. (B) Western blots show protein expression at 24 hai. Shown are representative images or blots from four independent biological replicates. (TIF) [file ppat.1005609.s006.tif]

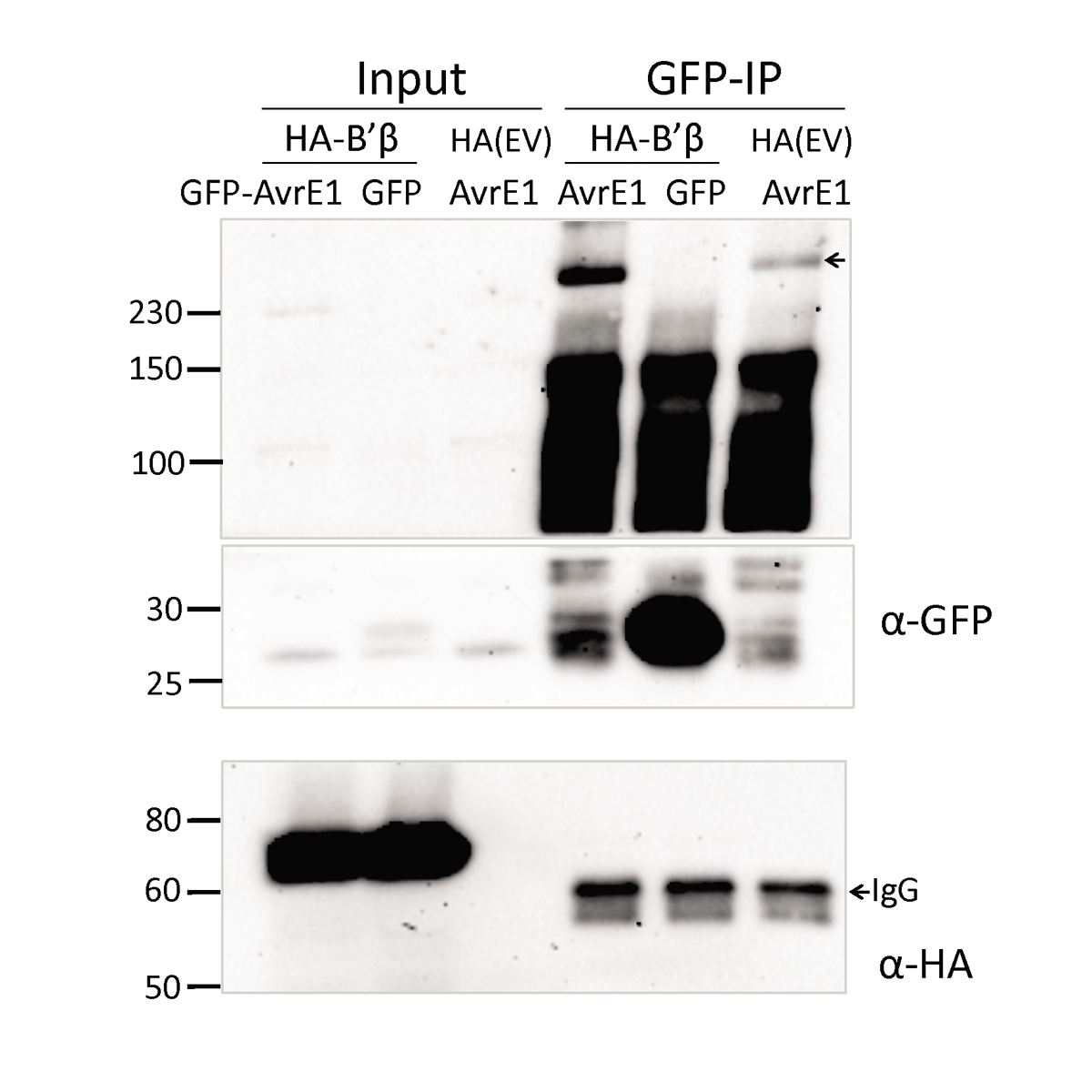

Supplement: S7 Fig — 35S::GFP-AvrE1 was co-expressed with 35S::HA-B’β in the presence of 1 μM cantharidin in N. benthamiana following Agro-transient expression. Co-immunoprecipitation of HA-B’β was tested using anti-HA antibody following GFP-pull down of full-length AvrE1. Shown is a representative blot from two biological replicates. Arrow indicates predicted size of full-length GFP-AvrE1 fusion protein. (TIF) [file ppat.1005609.s007.tif]

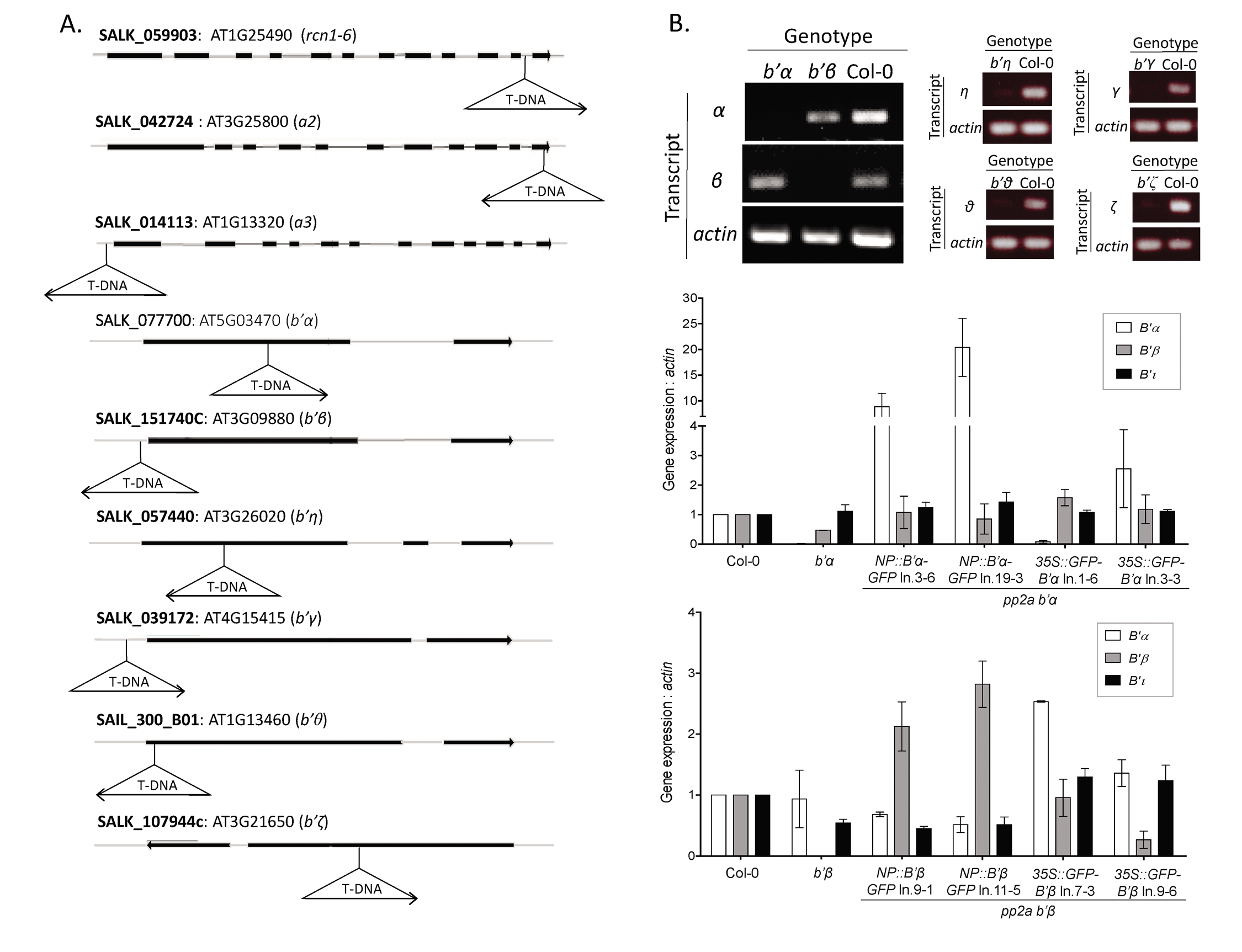

Supplement: S8 Fig — (A) Schematics of PP2A T-DNA insertion mutants used in this study. A subunit mutants: rcn1-6 and a2a3. B’ subunit mutants: α, β, η, ϒ, ϑ, and ζ. (B) Reverse transcription-PCR analysis of PP2A B’ subunit transcripts in different mutant backgrounds and complementation lines. cDNAs were generated from at least four independent plants of each genotype, and subjected to PCR or real-time PCR analysis using primers complementary to α, β, ι, η, ϒ, ϑ, ζ, and actin7 transcripts. (TIF) [file ppat.1005609.s008.tif]

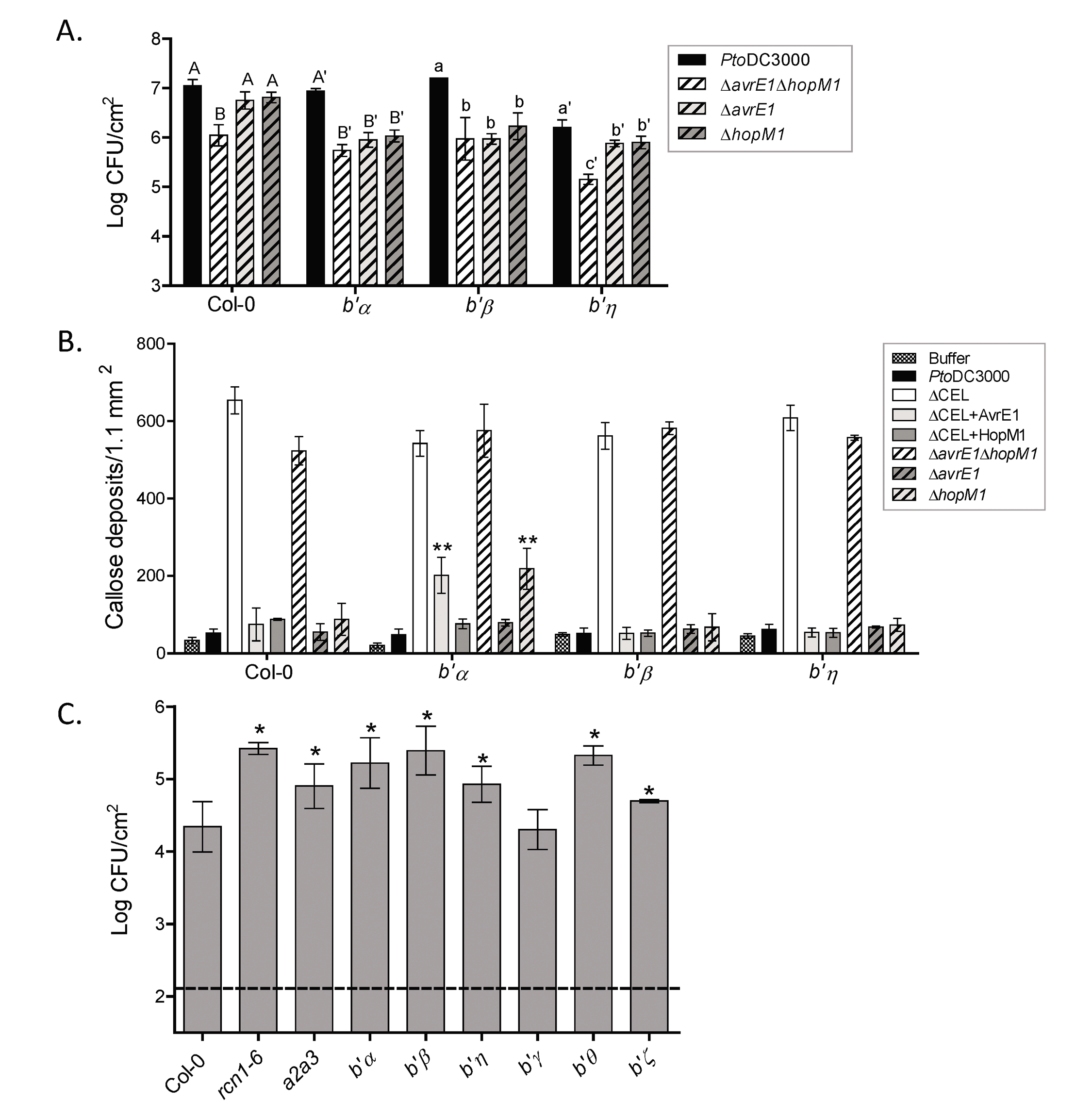

Supplement: S9 Fig — (A-B) Pto DC3000 mutant strains with deletions of AvrE1 and/or HopM1 exhibit comparable phenotypes to the corresponding plasmid complemented Pto ΔCEL strains. Similar to Pto ΔCEL, the double mutant Pto DC3000 strains, ΔavrE1ΔhopM1, grows less (A) and elicits more callose (B) than wild-type Pto DC3000 on wild-type Col-0 plants. Similarly, the single deletion mutant Pto DC3000 strains ΔhopM1 and ΔavrE1 also phenocopy ΔCEL+AvrE1 and ΔCEL+HopM1, respectively. (A) AvrE1 or HopM1, expressed in ΔhopM1 or ΔavrE1, respectively, failed to promote bacterial growth in Arabidopsis pp2a b’α and β mutants, but retained function in b’ η mutant. Leaves of five-week-old Arabidopsis plants were infiltrated with indicated bacterial strains (105 CFU/ml). Bacterial growth on different PP2A mutant plants was assayed four days after infiltration. Values shown are mean ± SEM from three biological replicates. Data was analyzed by one-way ANOVA followed by the Tukey test comparing different bacterial strains on individual plant genotypes. Different letters of the same style (eg. A vs B, or A’ vs B’) indicate a significant difference at P<0.05 within the same plant genotype. (B) AvrE1, expressed in ΔhopM1 just as in ΔCEL+AvrE1, was unable to fully suppress callose in the Arabidopsis pp2a b’ α mutant. Leaves of five-week-old Arabidopsis plants were infiltrated with the indicated bacterial strains (108 CFU/ml) or buffer (10 mM MgCl2), and collected at 16 hai for callose staining. Values shown are mean ± SEM from three biological replicates. ** indicates a significant difference by Student’s t-test at P<0.01. (C) Specific PP2A mutants are more susceptible to Pectobacterium carotovorum subsp. carotovorum (Pcc). Leaves of five-week-old Arabidopsis plants were infiltrated with wild-type Pcc (105 CFU/ml). Bacterial growth on different PP2A mutant plants was assayed four days after infiltration. Values shown are mean ± SD from three biological replicates. * indicates a statistically significant differ [file ppat.1005609.s009.tif]

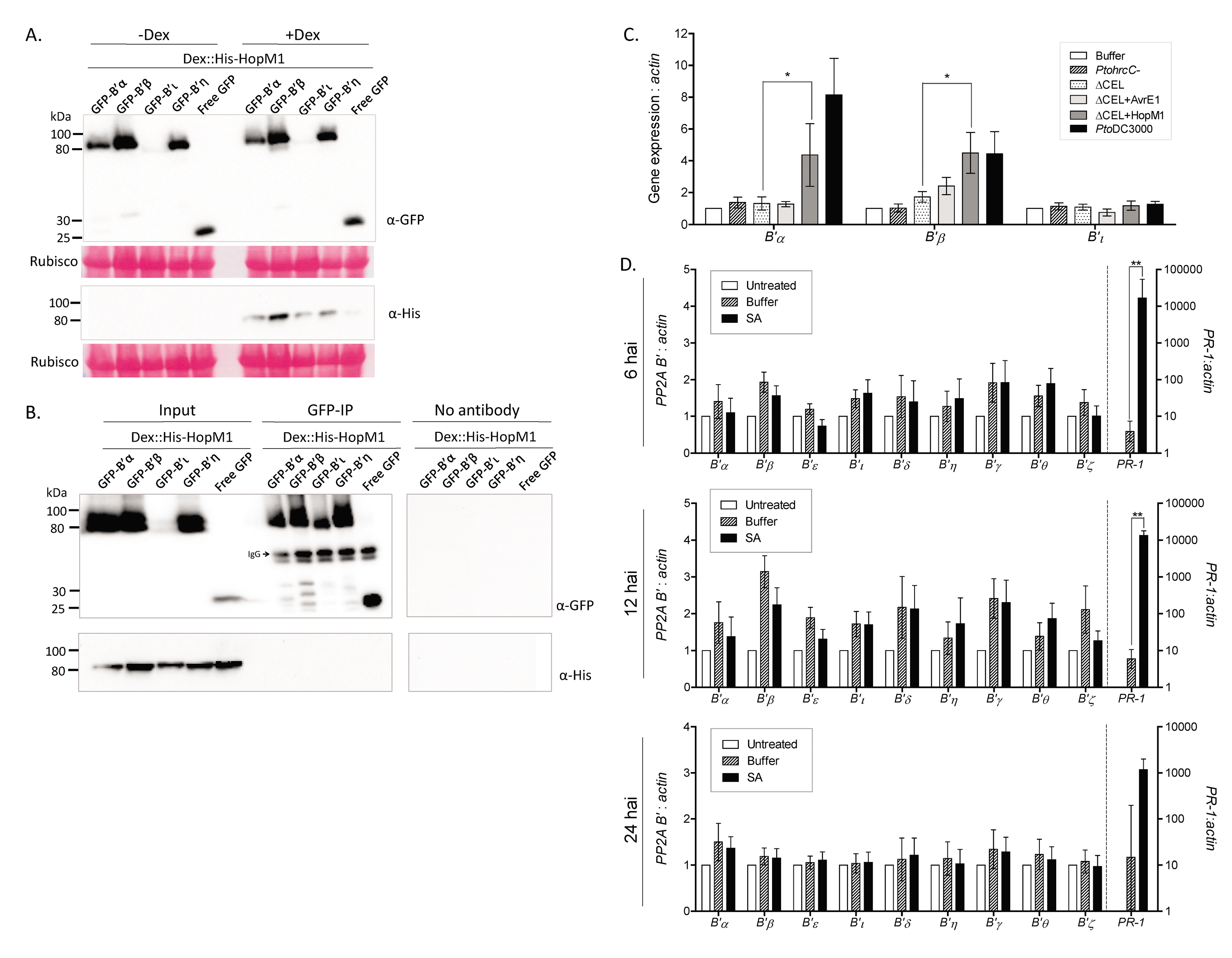

Supplement: S10 Fig — (A) HopM1 does not degrade PP2A B’ subunit proteins. At 48 hours following Agrobacterium-mediated transient expression in N. benthamiana, HopM1 expression was induced by spraying with 0.002% Silwet-77 with or without 30 μM Dex. At six hours after spraying, samples were collected for immunoblotting. (B) HopM1 does not detectably co-immunoprecipitate with PP2A B’ subunits. Samples prepared as in (A) were analyzed by co-immunoprecipitation, PP2A B’ subunit proteins were pulled-down with anti-GFP antibody and GFP-B’ subunits and His-HopM1 were detected by immunoblotting. Shown are representative blots from two biological replicates. (C) Transcripts of PP2A B’ α and β were induced by HopM1. Five-week-old Col-0 plants were infiltrated with indicated Pto strains at 108 CFU/ml and infiltrated leaves were collected at nine hai. Values shown are mean ± SEM from five to six biological replicates. * indicates a statistically significant difference by Student’s t-test at P<0.05. (D) Transcription of Arabidopsis PP2A B’ subunit genes are not affected by SA. Five-week-old Col-0 Arabidopsis leaves were sprayed with 500 μM SA (with 0.0002% Silwet-77) or buffer (H2O + 0.1% ethanol + 0.0002% Silwet-77). Samples were collected at 6, 12, and 24 hai and subjected to qRT-PCR. Values shown are mean ± SEM of normalized data from three biological replicates with transcript level in untreated samples set to 1. PP2A B’ genes are shown in regular scale, PR-1 transcript level is shown in log10 scale. Log transformed PR-1 transcript levels for untreated samples are 0, and are thus not shown on the graph. ** indicates a significant difference by Student’s t-test at P<0.01. (TIFF) [file ppat.1005609.s010.tiff]

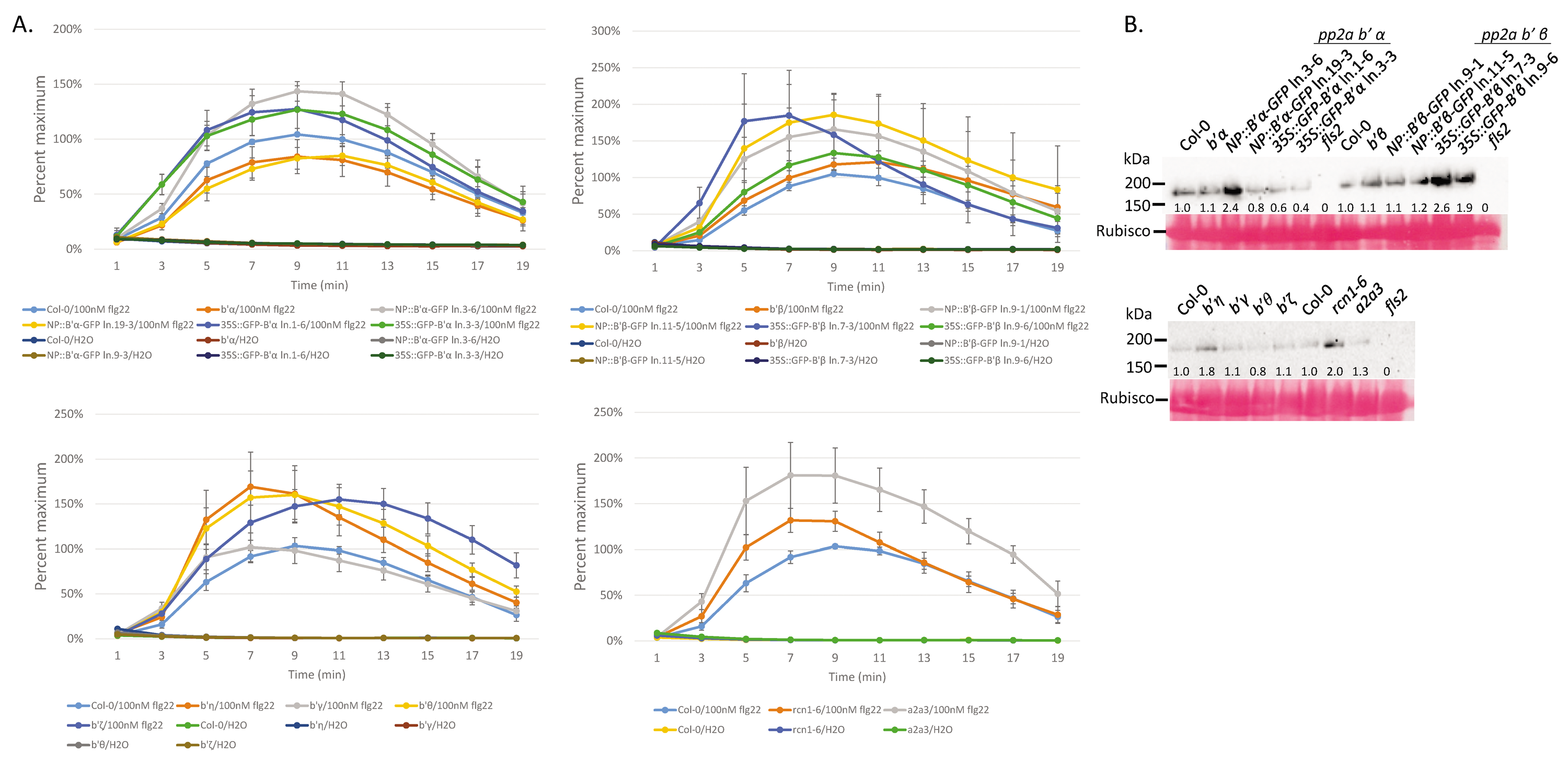

Supplement: S11 Fig — (A) The data shown was used to produce Fig 6. Leaf discs from five-week-old plants of the indicated genotypes were treated with 100 nM flg22 or H2O and ROS productions was monitored over time. Data was normalized within each biological replicate with the average value of the highest relative light unit (RLU) and the values preceding and following the peak value from the Col-0 flg22 treatment set to 100%. Shown are mean ± SEM from 3–6 biological replicates. (B). Steady-state FLS2 protein levels in PP2A mutants and complementation lines. Protein was extracted from untreated leaves from 4–6 individual plants of the same genotype and FLS2 protein was detected using polyclonal antibody against FLS2 [73]. Shown are representative blots from two biological replicates, numbers indicate average band intensity from both replicates with the band intensity of Col-0 set to 1. (TIF) [file ppat.1005609.s011.tif]

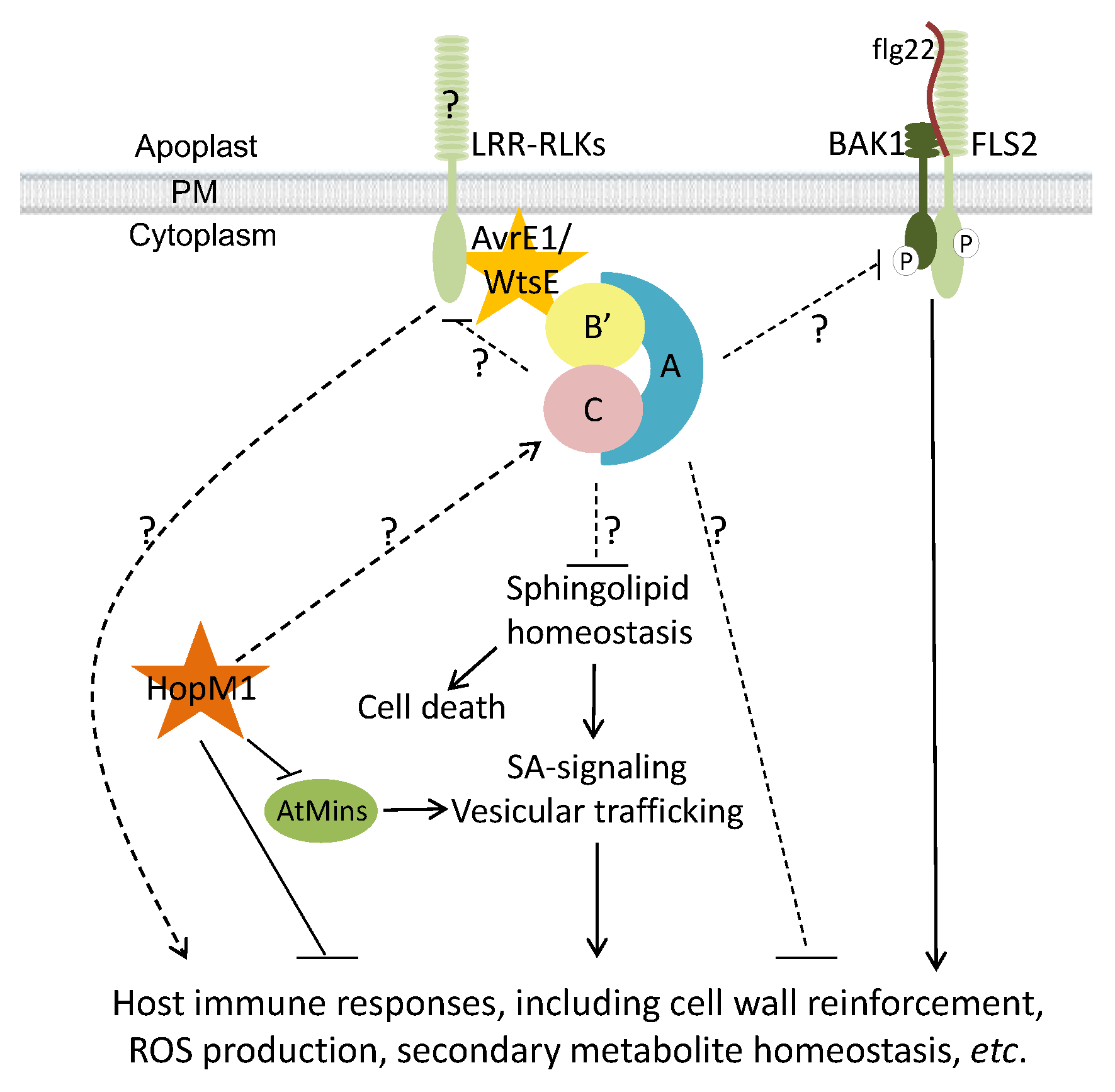

Supplement: S12 Fig — AvrE-family T3Es, including AvrE1 from Pseudomonas syringae pv. tomato, and WtsE from Pantoea stewartii pv. stewartii, associate with B’ subunits of host protein phosphatase 2A complexes to exploit their phosphatase activity. Potential mechanisms for modulation of plant immunity include: a. inhibition of known PAMP-recognition receptor complexes such as the FLS2/BAK1 complex; b. inhibition of other plasma-membrane localized leucine-rich repeat receptor-like complexes (e.g. those of WIP3, 4, and 5 and DIMP1-4 [41]) whose role in plant immunity are yet to be determined; c. disruption of host sphignolipid homeostasis with possible effects on SA-signaling, polarized transportation of antimicrobial compounds, and host cell death; d. disruption of other defense sectors, possibly including ethylene signaling and/or transcriptional regulation of metabolic enzymes. HopM1 also requires specific host PP2A complexes to promote virulence, possibly via indirect transcriptional modulation. Solid lines indicate established effects, dashed lines with question marks indicate hypothesized effects. (TIFF) [file ppat.1005609.s012.tiff]
