# Supplementary material for: Direct and Indirect Targeting of PP2A by Conserved Bacterial Type-III Effector Proteins
Source: PLoS Pathog. 2016 May 18;12(5):e1005609. doi: 10.1371/journal.ppat.1005609 (PMC4871590; doi:10.1371/journal.ppat.1005609)
Supplement: S2 Table — (PDF) [file ppat.1005609.s014.pdf]

Table S2. Primers used in this study.

Name	Sequence (5'-3')
**RT-PCR primers**	
B' alpha FP	5'-GATTGCTCGATGCCTCAGTAG-3'
B' alpha RP	5'-ACCGTGTACCGCTTGGTTCCA-3'
B' beta FP	5'- CACAGCCTGTCGAGTTCCAGC-3'
B' beta RP	5'-CCCACAATGTGCTCGTTGTTC-3'
B' iota FP	5'-AGAAGCAATGAGTGTGATGGAGT-3'
B' iota RP	5'-GTAGGATGGCTTGCCGGTTA-3'
B' delta FP	5'-CGCCAAATTGCTCAGTGCTT-3'
B' delta RP	5'-TCCAATGGCTGCTACCGTTT-3'
B' epsilon FP	5'-TGAGAGAGGAGCATAGGCTGT-3'
B' epsilon RP	5'-GCGGATCACAGTATCAGCCA-3'
B' eta FP	5'-TGCCTTAGACCTGGAGGAGG-3'
B' eta RP	5'-AGCCAGCTTGGTGTCAGTTT-3'
B' theta FP	5'-CCAAGTTGCTCGGTGTTTGAACAGT-3'
B' theta RP	5'-AGCTTGGTTCCAATGCTTCTGAGTG-3'
B' gamma FP	5'-TTCGTCAAATCGCTAGATGCCTCA-3'
B' gamma RP	5'-CGGGAACACAATGGGCATTATCAC-3'
B' zeta FP	5'-GCCGCTGAGTTTCAACGTTGTATGG-3'
B' zeta RP	5'-CACGCGTGTTTCTCTCAAGAGCTG-3'
PR-1 FP	5'-GAACATGTGGGTTAGCGAGAAG-3
PR-1 RP	5'-GTTCACATAATTCCCACGAGGA-3
actin7 FP	5'-CAGTGTCTGGATCGGAGGAT-3'
actin7 RP	5'-TGAACAATCGATGGACCTGA-3'
**Genotyping primers**	
rcn1-6 FP	5'-GTCAAGTACCTCACCTATAAC-3'
rcn1-6 RP	5'-CATAGCCAGCAACCAAAATGG-3'
a2 FP	5'-CTACCGAATGACCATTTTGCG-3'
a2 RP	5'-GCAAGACAATGGACAAAACCC-3'
a3 FP	5'-GTCTCATTTCTCGTTCTTCTG-3'
a3 RP	5'-CTCAACCCCTCCTACATACAG-3'
b' alpha FP	5'-CCGAGCGTCAATCCTTGTTC-3'
b' alpha RP	5'-TGAGGCATCGAGCAATCTGTT-3'
b' beta FP	5'-CGTGTACTCCGAGAAGATCTC-3'
b' beta RP	5'-CAGCAATTCTGAAGTTTCCTC-3'
b' eta FP	5'-GAGCTATTTGGCGAAACAGTG-3'
b' eta RP	5'-TCCAAAAGCACTGATAATGGC-3'
b' theta FP	5'-AAACTCAGCAATCCCATTGTG-3'
b' theta RP	5'-ATCGGAGGTGTTGTAATGTGC-3'
b' gamma FP	5'-TTGTCTCCAGGTTCAACAACC-3'
b' gamma RP	5'-AACACAGCCCTGCATGTAGAC-3'
b' zeta FP	5'-CACTCGTCGAAAAGAACTTGG-3'
b' zeta RP	5'-CCGAATCTCTTTATCGGGAAG-3'
LBa1 (Salk line T-DNA specific primer)	5'-TGGTTCACGTAGTGGGCCATCG-3'
LB1 (Sail line T-DNA specific primer)	5'-GCCTTTTCAGAAATGGATAAATAGCCTTGCTTCC-3'

Continued

Table S2 continued

Cloning primers		Restriction enzyme	Note
For pMDC43			
B’ alpha FP	5'-ACTAGTATGTTTAAGAAGATCATGAA -3'	SpeI	
B’ alpha RP	5'-AGGCCTCTAAGAAGTGATCATAGGAT -3'	StuI	
WIP2 FP	5'- CACCGACTAGTATGTGTGCCATCAACCTG -3'	SpeI	
WIP2 RP	5'- CACCGAGGCCTCTAGATCATGGGGCGATC -3'	StuI	
AvrE1-N’ FP (aa 1-898)	5'-ACTAGTGTGCAGTCACCATCGATCCA -3'	SpeI	
AvrE1-N’ RP	5'-GGATCCTTATGCGGTGTTTTTGTAGA -3'	BamHI	Stop codon added
AvrE1-M’ FP (aa 585-1400)	5'-ACTAGTGTGAACCTGACCAATGCGCT -3'	SpeI	start codon added
AvrE1-M’ RP	5'-GGATCCTTAGGGGTCGGCATTCGGGT -3'	BamHI	Stop codon added
AvrE1-C’ FP (aa 889-1795)	5'-ACTAGTGTGATCCGCGCCAACATCTA -3'	SpeI	start codon added
AvrE1-C’ RP	5'-GGATCCTTAGCTCTTCAGTTCGAACC -3'	BamHI	
AvrE1 full length FP	5'-ACTAGTGTGCAGTCACCATCGATCCA -3'	SpeI	
AvrE1 full length RP	5'-GGATCCTTAGCTCTTCAGTTCGAACC -3'	BamHI	
AvrE1-C’ k1k2 FP (KK1787-88AA)	5'-ACTAGTGTGATCCGCGCCAACATCTA -3'	SpeI	start codon added
AvrE1-C’ k1k2 RP	5'-TACCGAGCTCTTAGCTCTTCAGTTCGAACCCCTCTG CCGCCAAGTCGCCAGCC-3'	SacI	
HopQ1-1 FP	5'-TCGCCCTTCTGCAGACTAGTATGCATCGTCCTATCA CCGC-3'		
HopQ1-1 RP	5'-CCTTAGGCCTGAGCTCATCTGGGGCTACCGTCGAC-3'		
For pCsVMV-HA3-N-1300			
AvrE1-N’ FP (aa 1-898)	5'-GCTAGTCTAGAATGCAGTCACCATCGATC -3'	XbaI	
AvrE1-N’ RP	5'-CACGCGGATCCTGCGGTGTTTTTGTAGAT -3'	BamHI	
AvrE1-M’ FP (aa 585-1400)	5'-GCTAGTCTAGAATGAACCTGACCAATGCG -3'	XbaI	start codon added
AvrE1-M’ RP	5'-CACGCGGATCCGGGGTCGGCATTCGGGTC -3'	BamHI	
AvrE1-C’ FP (aa 889-1795)	5'-GCTAGTCTAGAATGATCCGCGCCAACATC -3'	XbaI	start codon added
AvrE1-C’ RP	5'-CACGCGGATCCGCTCTTCAGTTCGAACCC -3'	BamHI	Stop codon excluded
AvrE1-C’ k1k2 FP (KK1787-88AA)	5'-GCTAGTCTAGAATGATCCGCGCCAACATC -3'	XbaI	start codon added
AvrE1-C’ k1k2 RP	5'-TACCGGATCCGCTCTTCAGTTCGAACCCCTCT GCCGCCAAGTCGCCAGCC -3'	BamHI	Stop codon excluded
Complementation constructs			
B’ alpha FP	5'-CATGCAGTCGACTCCTTGGAATTTTGATGGGAAGA -3'	SalI	
B’ alpha RP	5'-CATGCAGCGGCCGCAGAAGTGATCATAGGATCTTC -3'	NotI	Stop codon excluded
B’ beta FP	5'-CATGCAGTCGACATAGTCTCCTACTCCATTACA -3'	SalI	
B’ beta RP	5'-CATGCAGCGGCCGCGGAAGTGATCATATGATCTTC -3'	NotI	Stop codon excluded
