# Supplementary material for: Direct and Indirect Targeting of PP2A by Conserved Bacterial Type-III Effector Proteins
Source: PLoS Pathog. 2016 May 18;12(5):e1005609. doi: 10.1371/journal.ppat.1005609 (PMC4871590; doi:10.1371/journal.ppat.1005609)
Supplement: S1 Text — (PDF) [file ppat.1005609.s015.pdf]

Supplemental Materials and Methods

Pnss growth assay in the presence or absence of cantharidin

Six-day-old maize seedlings were vacuum infiltrated with 10^9 CFU/mL of wild-type *Pantoea stewartii* subsp. *stewartii* (*Pnss* WT) or a *wtsE* mutant strain (*Pnss* WtsE-) supplemented with 0 or 50 µM cantharidin. Six leaf discs (8 mm diameter) per treatment were excised from the first true leaf of six individual infiltrated seedlings. Leaf discs were separated into three replicates with two discs per replicate and homogenized in 10 mM KPO_4 (PH 7.2) buffer. Bacterial titer was determined by plating serial dilutions and calculating CFU per cm^2 of infiltrated leaf. Statistical analysis on composite data from three biological replicates was carried out using one-way ANOVA followed by the Tukey HSD test ($P<0.05$) using SAS software.

Electrolyte leakage assays

For *Nicotiana benthamiana* electrolyte leakage assay, samples were collected from the infiltrated area at 16 hai to allow time for T-DNA transfer and gene expression. Twelve leaf discs were excised using a cork borer (diameter=8 mm), and were then floated in 15 ml of Milli-Q water. Conductivity was measured over time using a WTW model Cond 330i conductivity meter with a TetraCon 325 probe (WTW, Weilheim, Germany). Samples were not washed before measurement and damage during leaf disc excision thus contributes to the basal level electrolyte leakage at 16 hai. Conductivity per cm^2 of leaf area was calculated. Shown in S6C Fig are normalized values with buffer infiltrated samples subtracted from values for *Agrobaterium*-

inoculated samples. S6D Fig shows relative values representing: GFP-AvrE1$_{reading}$ / total area – free GFP$_{reading}$ / total area.

For Arabidopsis, eight leaf discs were excised using a cork borer (diameter=8 mm) right after infiltration, and washed in 25 ml Milli-Q water for 10 min. The wash water was then replaced with 10 ml fresh Milli-Q water, and conductance was measured over time using the conductivity meter. Conductivity per cm^2 of leaf area was calculated, and values for buffer infiltrated samples were subtracted from values for bacterial inoculated samples.

Total protein extraction and microsomal fractionation

Total protein was extracted by grinding 0.1 g of *N. benthamiana* or Arabidopsis tissue in 100 µl extraction buffer [20 mM Tris-HCl pH 7.5, 150 mM NaCl, 1 mM EDTA, 1% Triton X-100, 0.1% SDS, plant protease inhibitor cocktail (Sigma-Aldrich), 5 mM DTT]. Insoluble debris was pelleted by centrifugation at 20,000 x g for 10 min at 4 °C. Protein concentration was determined using the Bio-Rad protein assay reagents. Microsomal proteins were fractionated by grinding 0.1 g of tissue in 1 mL of buffer [10 mM Tris-HCl pH 7.0, 0.33 M Sucrose, 1 mM EDTA, plant protease inhibitor cocktail (Sigma-Aldrich), 5 mM DTT]. After centrifugation at 20,000 x g for 20 min at 4 °C to pellet the insoluble debris, the supernatant was the total fraction (T). Fifteen µL of 1 M $CaCl_2$ was added to 900 µL of the total fraction, incubated on ice for 1 hour, and the microsomal membranes were pelleted at 25,000 x g for 90 min at 4°C. The supernatant from this centrifugation was the soluble fraction (S) and the pellet was resuspended in 30 µL of 1x SDS-PAGE loading buffer and heated for 15 min at 65°C to form the microsomal fraction (M).

Bacterial strain construction, DNA manipulation, and generation of transgenic plants

The *Pto* ΔCEL + WtsE strain was constructed by transforming a pJH082 plasmid (pDSK519 carrying a 6.4-kb *Eco*RI/*Bam*HI fragment including the *wtsEF* operon and its native promoter [1]) into the *Pto* ΔCEL mutant strain via electroporation. Positive colonies were selected and maintained on KB plates containing rifampicin (75 µg/ml), spectinomycin (10 µg/ml for the ΔCEL deletion), and kanamycin (50 µg/ml, for selection of the pJH082 plasmid).

Arabidopsis *PP2A B'α* was PCR amplified using cDNA from Col-0 as template. The PCR product was cloned into pGEM-T Easy vector (Promega) by TA cloning for sequencing, and subsequently introduced into a gateway entry vector pCRCCDF at the *Spe*I/*Stu*I sites by restriction enzyme digestion. G-clones of Arabidopsis *PP2A B'β* (G19500), ξ (G61948), *ι* (G10648), and *η* (G12213) were obtained from the Arabidopsis Biological Resource Center. Entry clones bearing PP2A B' genes were then cloned into pMDC43 [2] by Gateway™ recombination (Invitrogen) to create *35S::GFP-B'* fusion constructs, or into pEARLEYGATE201 [3] vector to create *35S::HA-B'* fusion constructs. BiFC constructs were made by three-way Gateway™ recombination using pCRCCDF bearing inserts (or G-clones) with *35S::nGFP-pDONRp4p1*/*35S::cGFP-pDONRp4p1* and pH7m24GW [4] to obtain *35S::n/cGFP-PP2A B'* constructs. All constructs were transformed into *Agrobacterium tumefaciens* strain GV3101 by heat shock.

Maize *WIP1* full-length cDNA clone (ZM_BFc0189D23, cDNA in pCMV.SPORT-6.1) was obtained from Arizona Genomics Institute. The *Eco*RI/*Not*I fragment containing the *WIP1* insert was excised from the cDNA clone and inserted into the *Kpn*I/*Not*I sites in pCRCCDF entry vector. Maize

WIP2 full-length cDNA was PCR amplified using the 5'-RACE product as template. The PCR product was cloned into pGEM-T Easy vector by TA cloning for sequencing, and subsequently cloned into the *Spe*I/*Stu*I sites in pCRCCDF entry vector. *WIP1/WIP2-pCRCCDF* constructs were cloned into pMDC43 by LR recombination to create *35S::GFP-WIPs* fusion constructs. The final constructs were transformed into *A. tumefaciens* strain GV3101 by heat shock.

AvrE1 constructs (full-length AvrE1, aa 1-1795; AvrE1-N', aa 1-898; AvrE1-M', aa 585-1400; AvrE1-C', aa 889-1795; and AvrE1-C' k1k2 mutant, aa 888-1795, KK1787-88AA) were PCR amplified using AvrE1-pUCP19 plasmid [5] as template. The PCR products were either cloned directly into the *Xba*I/*Bam*HI sites on pCsVMV-HA3-N-1300 to create *CsVMV::AvrE1-HA* fusion constructs or the PCR products were first cloned into pGEM-T Easy vector by TA cloning for sequencing. The AvrE1-C' k1k2 construct was then cloned into the SpeI/*Sac*I sites on pCRCCDF. Other AvrE1 constructs were cloned into the SpeI/*Stu*I sites on pCRCCDF by filling in the C-terminal *Bam*HI cohesive end to create a blunt end. All AvrE1 constructs were then introduced into either pMDC43 by LR recombination to create *35S::GFP-AvrE1* fusion constructs or into BiFC vectors using same approach described above. All constructs were transformed into *A. tumefaciens* strain GV3101 by heat shock.

HopQ1-1 was PCR amplified using *Pto* DC3000 genomic DNA as template. The PCR product was introduced into pCRCCDF entry vector between the *Spe*I/*Sac*I sites using Gibson assembly® (NEB, E2611S), sequenced, and subsequently cloned into pMDC43 by LR recombination to create

35S::GFP-HopQ1-1 fusion construct. The final construct was transformed into *A. tumefaciens* strain GV3101 by heat shock.

For native promoter driven PP2A B' complementation constructs, Col-0 genomic DNA was used as a template to PCR amplify *B'α* (including 2.2 kb upstream of the translation start site), and *B'β* (including 1.5 kb upstream of translation start site). The PCR products were introduced into the Gateway entry vector pENTR1A (Invitrogen) at the *Sal*I/*Not*I sites, sequenced, and then into the promoter assay binary vector pHGWFS7 [6] by LR recombination. Both native promoter driven PP2A B' complementation constructs and the *35S::GFP-PP2A B'* constructs in pMDC43 were introduced into *A. tumefaciens* strain GV3101 by heat shock and then transformed into the respective Arabidopsis *pp2a b'* mutants (namely *α* and *β*) by the floral dip method [7]. Transgenic progeny were selected by growth on Gamborg's B5 (Invitrogen) agar plates containing 20 µM hygromycin B (Sigma-Aldrich). Independent lines with single insertion loci, as determined by 3:1 segregation of hygromycin B resistance in the T2 generation, were selected and propagated to homozygosity.

Maize protein phosphatase 2A B' subunit proteins prediction and protein phylogeny analysis.

Seven maize ESTs (BG320458, AI621449, AI586835, AW165473, AW562969, AW052973, and AW600513) were previously predicted to encode for PP2A B' proteins [8]. The seven ESTs correlate with six unique maize proteins (NP_001169040, NP_001146864, NP_001183245, NP_001146050, NP_001141673, NP_001148974), all of which contain the B56 domain that is conserved in the PP2A B' subunit family. Additional B' proteins were identified using the BlastP

algorithm on NCBI (http://blast.ncbi.nlm.nih.gov/) using WIP1 (NP_001146864) and WIP2 (NP_001148974) as queries to search for homologous proteins against the maize protein database. Three additional proteins containing B56 domain were identified (NP_001105904, AFW77313, AFW74402). The six proteins predicted based on ESTs were also identified using the latter approach.

Protein sequences of all nine Arabidopsis PP2A B' proteins and the nine identified maize PP2A B' proteins were aligned by MUSCLE with ambiguous regions removed using Gblocks. The tree was generated by the maximum likelihood method using PhyML with reliability of internal branches determined by the aLRT test. Software used: phylogeny.fr/version2_cgi/index.cgi.

Supplemental References:

1. Ham JH, Majerczak D, Ewert S, Sreerekha MV, Mackey D, Coplin D. WtsE, an AvrE-family type III effector protein of *Pantoea stewartii* subsp. *stewartii*, causes cell death in non-host plants. Mol Plant Pathol. 2008;9(5):633-643.
2. Curtis MD, Grossniklaus U. A gateway cloning vector set for high-throughput functional analysis of genes *in planta*. Plant Physiol. 2003;133(2):462-469.
3. Earley KW, Haag JR, Pontes O, Opper K, Juehne T, Song K, et al. Gateway-compatible vectors for plant functional genomics and proteomics. The Plant journal : for cell and molecular biology. 2006;45(4):616-629.
4. Boruc J, Van den Daele H, Hollunder J, Rombauts S, Mylle E, Hilson P, et al. Functional modules in the Arabidopsis core cell cycle binary protein-protein interaction network. Plant Cell. 2010;22(4):1264-1280.
5. DebRoy S, Thilmony R, Kwack YB, Nomura K, He SY. A family of conserved bacterial effectors inhibits salicylic acid-mediated basal immunity and promotes disease necrosis in plants. Proc Natl Acad Sci USA. 2004;101(26):9927-9932.
6. Karimi M, Inze D, Depicker A. GATEWAY vectors for Agrobacterium-mediated plant transformation. Trends Plant Sci. 2002;7(5):193-195.
7. Clough SJ, Bent AF. Floral dip: a simplified method for *Agrobacterium*-mediated transformation of *Arabidopsis thaliana*. Plant J. 1998;16(6):735-743.
8. Terol J, Bargues M, Carrasco P, Perez-Alonso M, Paricio N. Molecular characterization and evolution of the protein phosphatase 2A B' regulatory subunit family in plants. Plant Physiol. 2002;129(2):808-822.
